# Adaptive DNA amplification of synthetic gene circuit opens a way to overcome cancer chemoresistance

**DOI:** 10.1073/pnas.2303114120

**Published:** 2023-11-29

**Authors:** Yiming Wan, Quanhua Mu, Rafał Krzysztoń, Joseph Cohen, Damiano Coraci, Christopher Helenek, Christopher Tompkins, Annie Lin, Kevin Farquhar, Erin Cross, Jiguang Wang, Gábor Balázsi

**Affiliations:** ^a^Department of Biomedical Engineering, Stony Brook University, Stony Brook, NY 11794; ^b^The Louis and Beatrice Laufer Center for Physical and Quantitative Biology, Stony Brook University, Stony Brook, NY 11794; ^c^Department of Chemical and Biological Engineering, Division of Life Science, State Key Laboratory of Molecular Neuroscience, The Hong Kong University of Science and Technology, Hong Kong Special Administrative Region 999077, China; ^d^KromaTid, Inc., Longmont, CO 80501; ^e^Stony Brook Cancer Center, Stony Brook University, Stony Brook, NY 11794

**Keywords:** drug resistance, evolution, RNA sequencing, synthetic biology, DNA amplification

## Abstract

Drug resistance continues to impede the success of cancer treatments, creating a need for experimental model systems that are broad, yet simple, to allow the identification of mechanisms and novel countermeasures applicable to many cancer types. To address these needs, we investigated a set of engineered mammalian cell lines with synthetic gene circuits integrated into their genome that evolved resistance to Puromycin. We identified DNA amplification as the mechanism underlying drug resistance in 4 out of 6 replicate populations. Triplex-forming oligonucleotide (TFO) treatment combined with Puromycin could efficiently suppress the growth of cell populations with DNA amplification. Similar observations in human cancer cell lines suggest that TFOs could be broadly applicable to mitigate drug resistance, one of the major difficulties in treating cancer.

## Significance

Cancers still resist treatment too often, creating a need for new insights into cancer drug resistance and new treatment options. Experimental models that are broad, yet simple could aid with the identification of mechanisms and development of countermeasures of drug resistance. Here, by studying engineered mammalian cells that previously evolved resistance to a drug, we found DNA amplification as the most common cause of drug resistance. A nucleotide treatment combined with the original drug efficiently suppressed the growth of hamster and cancer cells with DNA amplification, suggesting broadly applicable therapies that might combat chemoresistance in cancer.

Drug resistance impedes the success of cancer treatment, leading to relapse associated with more aggressive phenotypes, including cancer metastasis (1). Cancer cells can develop resistance to traditional and newer clinical approaches, such as targeted therapy (2) or immunotherapy (3). Despite some similarities to drug resistance of infectious bacteria, the mechanisms of cancer drug resistance are more complex, which relates to the general complexity of mammalian cells, tissues, and bodies compared to bacteria (4). Therefore, broadly mammalian, yet systematically controllable experimental model systems would be useful to eliminate some complexity while still maintaining general mammalian characteristics. Such model systems could help unravel and address generic, mammalian cellular capabilities relevant to cancer drug resistance.

Drug resistance in cancer emerges through cellular evolution, where selection pressure during treatment favors the spreading of increasingly resistant variants (5). Most drug resistance, whether intrinsic or acquired, was traditionally attributed to genetic mutations, or more recently, to epigenetic processes taking place in neoplastic cells (6). Still, a growing number of studies indicate that the biological mechanisms underlying the emergence and spreading of drug-resistant cancer cells are diverse, complex, and insufficiently understood (7–9). Analysis of clinical data or animal studies aims to unravel drug resistance in close association with human patients. Yet, the complexity of such in vivo systems compromises the systematic characterization and interpretation of adaptive changes in terms of their molecular causes and consequences. Thus, to complement in vivo studies, simple yet general in vitro model systems are needed for the systematic, mechanistic examination of individual aspects of mammalian drug resistance.

**Fig. 1.**
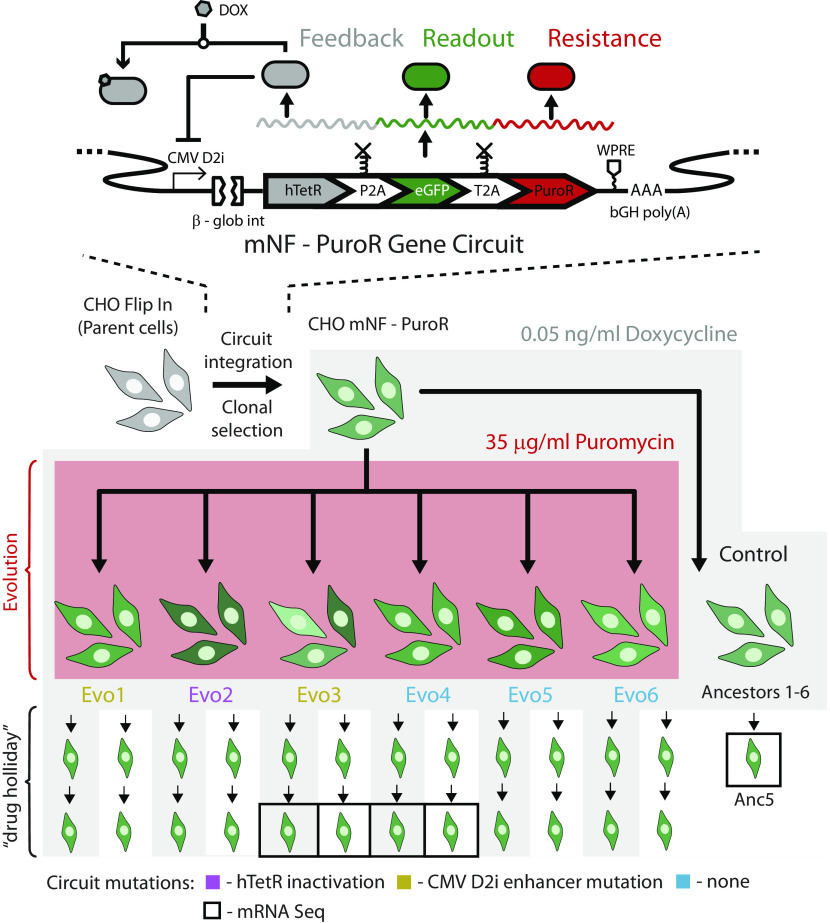
Schematic illustration of how the cell populations in this study were derived. Schematic illustration of the mNF gene circuit regulating the Puromycin resistance gene PuroR and the fluorescent reporter eGFP that was chromosomally integrated into monoclonal CHO cells and then evolved to develop stable Puromycin resistance. Each of the six replicate populations evolved independently in the same constant Puromycin concentration of 35 μg/mL until it fully recovered to confluence. We cultured and passaged each replicate subsequently in the absence of Puromycin, with or without 0.05 ng/mL Dox, with samples frozen right after evolution, and then repeatedly during a “drug holiday”. Sanger sequencing revealed hTetR protein-coding mutations in replicate Evo2, enhancer mutations in Evo1 and Evo3, while Evo4, Evo5, and Evo6 were devoid of gene circuit mutations. Replicates Evo3 and Evo4 representing the last two scenarios were selected for mRNA sequencing, in addition to ancestral replicate Anc5 used for control purposes.

As a novel approach to unravel the intricacies of drug resistance, synthetic biological systems provide a controlled framework for understanding cellular adaptation to drug treatment. Previously, we developed in vitro experimental systems to study the evolution of drug resistance in yeast (10, 11) and then mammalian cells (12). These model systems consist of cell lines carrying a stably integrated synthetic gene circuit controlling a drug resistance gene. When exposed to the drug that the gene’s protein product resists, such engineered cells reveal mechanisms of selection, survival, and long-term adaptation to drug treatment. For example, we evolved in various constant concentrations of Puromycin six replicate populations (Evo1 through Evo6) of originally monoclonal Chinese hamster ovary (CHO) cells with a chromosomally integrated mammalian negative feedback (mNF) synthetic gene circuit controlling the Puromycin resistance gene PuroR (Fig. 1). We showed that preexisting high PuroR expression variability promotes the evolution of drug resistance only in high stress, whereas it hinders resistance evolution in low stress (12). All mNF replicates evolved to resist Puromycin by increasing their PuroR expression compared to ancestral control populations that grew up without Puromycin (Fig. 1). Sanger sequencing indicated a protein-coding sequence mutation in one replicate population (Evo2), enhancer mutations in two populations (Evo1 and Evo3), and unchanged gene circuit sequences in three populations (Evo4, Evo5, and Evo6). Overall, the replicate-specific causes of elevated PuroR expression, and possible treatment options to counter these adaptive changes remain unknown.

Here, we used quantitative Polymerase Chain Reaction (qPCR), transcriptome sequencing (RNA-seq), and flow cytometry to characterize evolutionary changes at the DNA, messenger RNA (mRNA), and protein levels in evolved mNF cell populations. These experiments indicated DNA amplification as the most common mechanism of drug resistance, consistent with prior evidence for resistance-conferring (13) DNA amplification in advanced cancers (14, 15) on chromosomes or in extrachromosomal DNA (16–18). We show that DNA amplification enables the use of triplex-forming oligonucleotide (TFO) treatment, a recently proposed nucleotide-based anti-cancer therapy (19) to counter mammalian drug resistance with minimal side effects. We confirm these results in human cancer cell lines that are drug-resistant due to DNA amplification (20, 21). These findings could suggest a broadly applicable treatment strategy to mitigate cancer drug resistance in combination with chemotherapeutics or targeted therapies.

## Results

### RNA Sequencing Reveals Adaptive Overexpression of Gene Circuit Parts.

Negative feedback (NF) or linearizer gene circuits enable precise gene expression tunability, with low noise and linear dose–response to external inducer concentration in yeast (22) and mammalian cells (12, 23, 24). The mNF gene circuit (Fig. 1) consists of the humanized tetracycline repressor (hTetR) that binds to tetO2 sites within D2i, a modified CMV promoter, to repress the expression of various target genes as well as its own gene. The inducer Doxycycline (Dox) added to the growth medium renders hTetR unable to bind to D2i, causing gene expression to increase precisely, with low noise as inducer concentration increases. Using low-noise gene expression control by mNF, paired with another high-noise conferring gene circuit revealed how gene expression noise affects the adaptation of hamster cells grown in Puromycin (12), followed by a drug holiday (Fig. 1). Nonetheless, it remained unclear by what mechanisms the six replicate mNF populations increased enhanced Green Fluorescent Protein (eGFP) and thus PuroR expression (12) to become Puromycin-resistant.

**Fig. 2.**
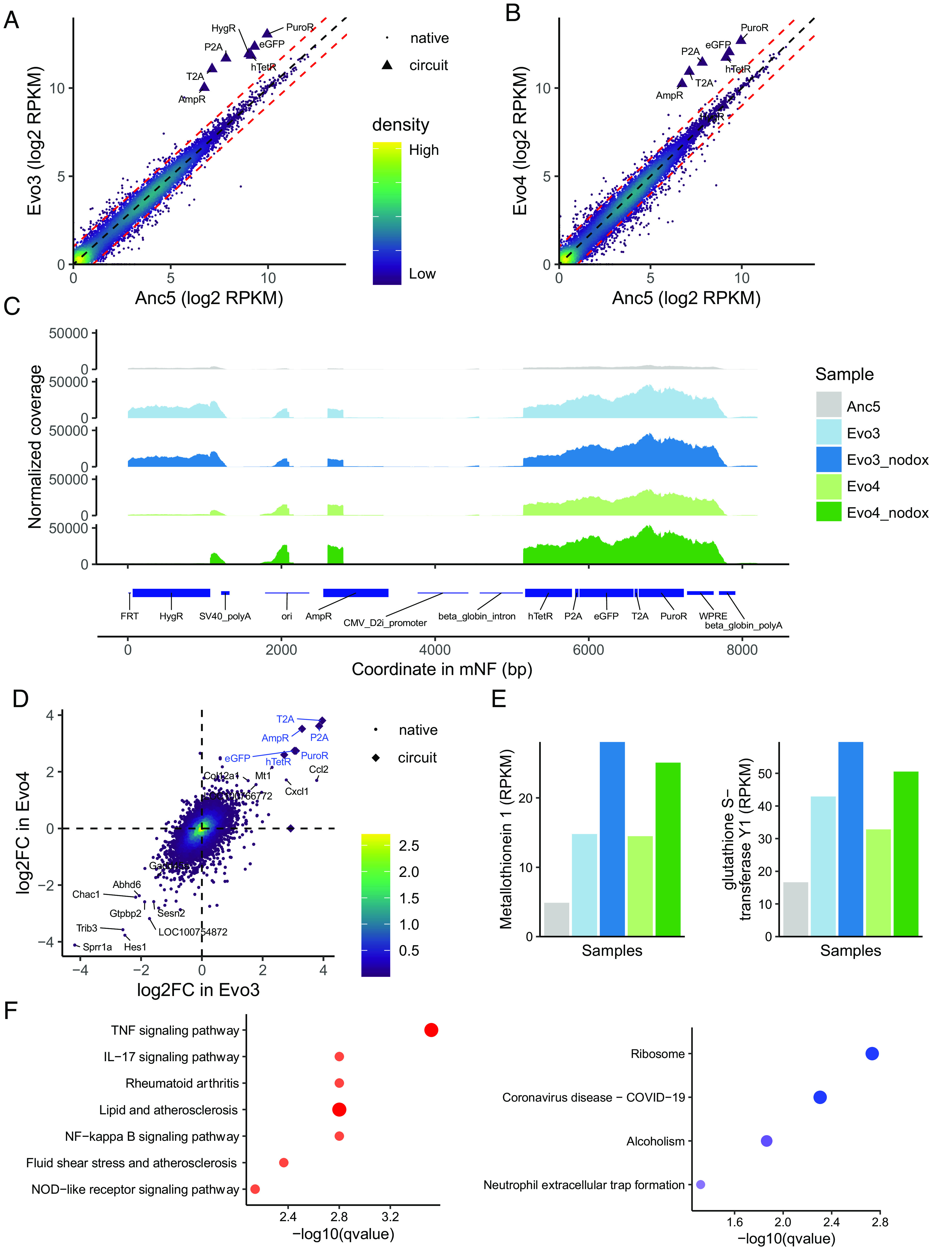
Transcriptomic changes after adaptation to Puromycin in replicates Evo3 and Evo4. (*A* and *B*) RNA sequencing shows more prominently elevated expression of RNAs mapping to gene circuit regions compared to native genes in Puromycin-resistant mNF populations Evo3 (*A*) and Evo4 (*B*) compared to the untreated control Anc5. (*C*) Normalized RNA sequencing coverage over the mNF gene circuit in the untreated control Anc5, as well as the Puromycin-resistant Evo3 and Evo4 replicate populations cultured with or without Dox. (*D*) Fold-changes of RNA expression levels for the native (circles) and circuit (diamonds) genes in the Puromycin-resistant Evo3 and Evo4 replicate populations versus the untreated control. Blue and black colors indicate significantly up-regulated and down-regulated circuit genes and native genes, respectively. (*E*) Overexpression of metallothionein 1 (*Mt1*, *Left*) and glutathione S-transferase Y1 (LOC100766772, *Right*) in the Puromycin-resistant Evo3 and Evo4 replicate populations cultured with or without Dox, compared to ancestral control Anc5. (*F*) Enriched KEGG pathways of the upregulated (*Left*) and downregulated (*Right*) genes. The sizes of the dots indicate the number of overlapping genes, while the depth of the colors indicates the ratio of the overlapping genes.

To find out how the evolved replicate mNF populations (Evo1 to Evo6) acquired Puromycin resistance, we asked how mRNA levels reflect the elevated PuroR protein levels of all six Puromycin-resistant mNF populations (12). Thus, we investigated the transcriptomes of evolved versus control mNF samples by Illumina RNA sequencing. Since in replicate Evo2, an hTetR coding mutation trivially explains elevated PuroR expression (12), we did not sequence this replicate. Instead, we selected two replicates representing the other two scenarios of evolution in 35 μg/mL Puromycin (12): i) Evo3, which, like Evo1, had a single-nucleotide polymorphism of unknown effect in the distal enhancer region of the modified CMV (D2i) promoter; and ii) Evo4, which, like Evo5 and Evo6, did not have any detectable point mutations within the mNF gene circuit sequence. We extracted RNA from Dox-induced and uninduced Evo3 and Evo4 samples frozen at passage 3 during the drug holiday; and from the ancestral mNF replicate 5 (Anc5) exposed to 0 μg/mL Puromycin (no Puromycin) for control purposes (Fig. 1). Anc5 is genetically equivalent to all other ancestor populations (Anc1 to Anc6), which were grown to confluence without passaging (12). We aligned the resulting reads from each sample to the Chinese hamster (*Cricetulus griseus* CriGri-PICRH-1.0, GCF_003668045.1) reference genome (25, 26) complemented with the synthetic gene circuit sequence (*Materials and Methods*). The analysis confirmed that PuroR mRNA levels were much higher (fivefold to 10-fold) in all evolved replicate populations compared to the ancestral control, consistent with the elevated post-evolution PuroR protein levels (Fig. 2*A* and *SI Appendix*, Fig. S1*A*). Additionally, eGFP and hTetR transcript levels were also elevated except for introns and poly-A tails (*SI Appendix*, Fig. S1 *B* and *C*). PuroR, hTetR, and eGFP expression levels were correlated across replicate populations, as expected due to the co-expression of these gene circuit components. More surprisingly, in Evo3 we also observed elevated expression of transcripts mapping to the HygroR selection marker, despite the lack of Hygromycin selection during or after the evolution experiments (Fig. 2*C*). Finally, we also observed high expression of transcripts mapping to some other plasmid backbone regions such as AmpR and oriC (Fig. 2*C*). Overall, RNA sequencing indicated highly elevated transcription of all mNF gene circuit components, and unexpectedly, other plasmid elements integrated into the CHO genome.

Seeking native transcriptome changes that might contribute to acquired Puromycin resistance, we also examined the levels and sequences of *C. griseus* transcripts. Multiple native transcripts were significantly over- or underexpressed in the evolved replicate populations, although native expression changes were less prominent than those of the gene circuit components (Fig. 2*D*). For example, the overexpressed metallothionein gene *Mt1* (27) and glutathione S-transferase Y1 (LOC100766772) mediate metal homeostasis and heavy metal detoxification and could protect cells from oxidative stress and hydroxyl radicals. This indicates their involvement in general stress response (28), possibly including stress associated with Puromycin treatment (Fig. 2*E*). Likewise, the upregulation of cytokines and chemokines such as Ccl2 and Cxcl1 might indicate stress response upon Puromycin treatment (Fig. 2*D*). On the other hand, the underexpression of Trib3, a negative NF-kappaB regulator could desensitize cells to apoptosis (Fig. 2*D*). Concordantly, functional enrichment analysis revealed significant enrichment of TNF and NF-kappaB signaling among upregulated genes, while the ribosome pathway was enriched among down-regulated genes (Fig. 2*F*). Taken together, the expression patterns of the native genes reflected the avoidance of death, as well as the elevated stress response of CHO cells to Puromycin. While native genes involved in detoxification might offer some protection, increased expression of gene circuit components is most prominent, and directly explains the evolved resistance to Puromycin.

### qPCR Indicates DNA Amplification of PuroR and Other Circuit Components.

**Fig. 3.**
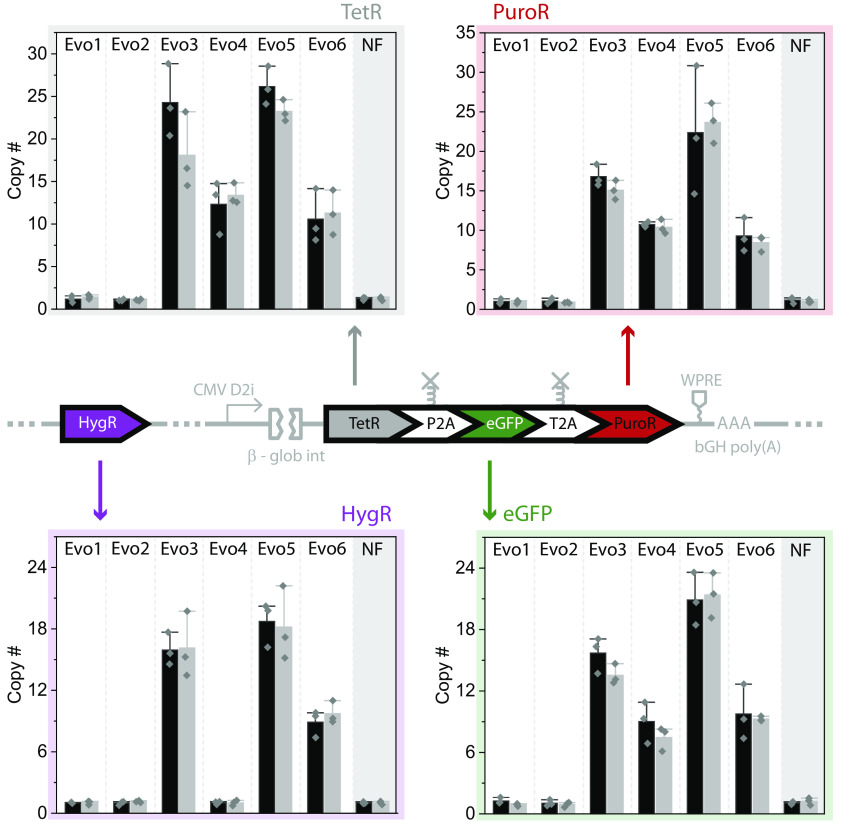
The four mutation-free evolved replicates have mNF gene circuit DNA amplification. Copy number analysis of PuroR (*Top* *Right*), hTetR (*Top* *Left*), eGFP (*Bottom* *Right*), and HygR (*Bottom* *Left*) genes shows consistently elevated DNA content in experimental replicates Evo3 through Evo6 compared to untreated mNF control population Anc5. The HygR gene amplification is not presented in Evo4, which is consistent with the RNA sequencing data. Evo2 lacks DNA amplification as expected due to the hTetR coding mutation. The survival mechanism of Evo1 that also lacks DNA amplification is unclear. The DNA copy number of gene circuit components is normalized to internal reference gene Vinculin, Mean ± SD of three independent repeats.

The universally strong upregulation of mNF circuit genes and other plasmid components suggests that the gene circuit might have undergone DNA amplification (29). To test whether DNA amplification underlies highly elevated transcription of mNF gene circuit components and various integrated plasmid elements, we performed copy number quantification on genomic DNA from mNF experimental replicates Evo1 through Evo6 compared to untreated control Anc5. We designed primers to amplify the mNF circuit genes PuroR, hTetR, and eGFP as well as the CHO-native control gene Vinculin. After we verified primer specificity and amplification efficiency with CHO genomic DNA, we performed qPCR to determine the relative copy numbers for all evolved replicates (Evo1 through Evo6) versus Anc5. These assays indicated PuroR, hTetR, and eGFP DNA amplification in replicates Evo3 through Evo6, but not Evo1 and Evo2, compared to Anc5 (Fig. 3).

Likewise, qPCR assays confirmed that HygroR DNA amplification, which was present in Evo3, but not Evo4 (Fig. 3). This is also consistent with HygroR mRNA overexpression only in Evo3, but not Evo4 observed by RNA-seq. Similar qPCR assays also identified HygroR amplification in Evo5 and Evo6, but not in Evo1 or Evo2 (Fig. 3).

To visualize DNA amplification, we partnered with KromaTid, Inc. to conduct DNA fluorescence in situ hybridization using a custom directional Genomic Hybridization™ (dGH) in-site assay for each evolved sample, as well as the mNF control (*Materials and Methods*). We observed mNF DNA amplification in Evo3 to Evo6, but not in Evo1 and Evo2 compared to the control sample (*SI Appendix*, Fig. S2*A*). Whereas some DNA amplification occurred as extracellular DNA (ecDNA), especially in Evo3 (*SI Appendix*, Fig. S2*B*), tandem mNF repetition was most common in Evo3 to Evo6.

Overall, this analysis confirmed DNA amplification as the source of elevated mRNA expression of gene circuit components in replicates Evo3 through Evo6, which lacked intra-circuit mutations. As expected, there was no DNA amplification in replicate Evo2, which harbors an hTetR-disabling mutation. Somewhat surprisingly, there was DNA amplification in Evo3 but not Evo1, although both post-evolution populations contained at least some variants with the same gene circuit mutation, located within the enhancer of the modified CMV promoter D2i.

### Protein-Level Sensors Indicate hTetR Repressor Activity in Most Evolved Clones.

**Fig. 4.**
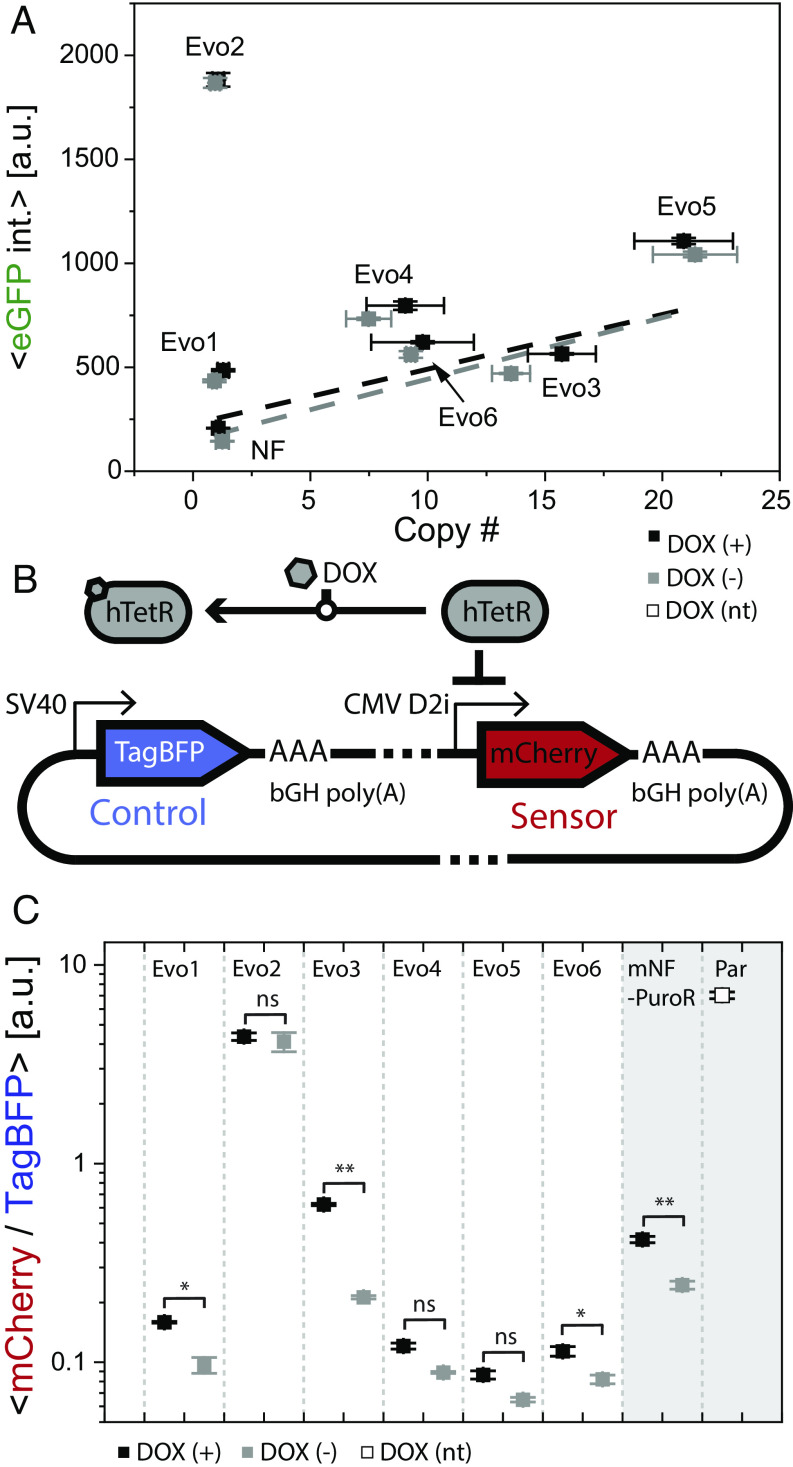
The hTetR protein remains active in the evolved replicates. (*A*) Average eGFP intensity per cell versus DNA copy number of the eGFP ORF in each evolved replicate population. High DNA copy number is associated with higher eGFP intensity, with a linear trend for both Dox-treated and untreated cells (Pearson's r of 0.85 and 0.81 respectively) after excluding the Evo2 population with inactive hTetR. Dashed lines indicate the linear fit to the Dox-treated and untreated data points. (*B*) Schematic illustration of the hTetR activity-sensing plasmid. mCherry is driven by an hTetR-dependent promoter (CMV-D2i) whereas TagBFP is constitutively expressed and used as a plasmid copy number control in each transfected cell. (*C*) Average relative mCherry/BFP fluorescence values (mCherry expression normalized by BFP expression to estimate plasmid copies per cell. NF: Anc5 cells; Par: parental CHO cells without a gene circuit. Unpaired *t* test was performed for each comparison, *P < 0.05, **P < 0.01, ***P < 0.001, ****P < 0.0001.

Having evidence of DNA amplification as the source of elevated mRNA expression in most evolved replicate populations does not yet address the question whether all circuit protein levels are also elevated. To investigate protein level changes, we set out to confirm that eGFP expression levels were elevated after the evolution experiments, as we observed previously (12). We measured by flow cytometry the eGFP fluorescence intensity of each evolved replicate population as well as the control replicate population Anc5 without Puromycin, in the same Dox concentration (0.05 ng/mL) in which they evolved, as well as without Dox. We observed significantly elevated eGFP fluorescence intensity in all Evo replicate populations compared to Anc5 (Fig. 4*A*), confirming the earlier results. After excluding Evo1 and Evo2 whose resistance was independent of DNA amplification, eGFP intensity in Evo3 to Evo6 correlated positively with DNA copy numbers, indicating a direct protein-level relevance of DNA amplification and gene circuit expression.

Considering that hTetR is the regulator that controls all expression levels in the mNF gene circuit, we asked whether hTetR protein activity is elevated consistently with the higher hTetR mRNA and DNA levels in most replicate populations. To quantify the hTetR protein level in CHO replicates, we developed a hTetR activity sensor vector that contains the reporter mCherry expressed directly from the CMV-D2i promoter, which can be repressed by hTetR binding to two operator sites (24). Meanwhile, we added a constitutively expressed BFP reporter onto the plasmid backbone as an internal reference to report on plasmid copy numbers (Fig. 4*B*). This allows normalizing mCherry expression by BFP expression, to obtain an mCherry/BFP ratio that serves as an inverse measure of free (active) hTetR protein levels. Specifically, the mCherry/BFP ratio should be lower when active hTetR protein level is higher. Therefore, transient transfection of this sensor into CHO replicates provides a measure for free (active) hTetR protein presence by the mCherry/BFP ratio.

To test the activity of hTetR in various evolved replicate populations, we transfected the hTetR activity sensor into all evolved replicates and measured the mCherry/BFP ratio. Compared to the mNF control population, we observed higher mCherry/BFP ratio indicating lower hTetR activity only in Evo2, with or without Dox (Fig. 4*C*). Evo1, Evo4, Evo5, and Evo6 had lower mCherry/BFP ratios indicating higher hTetR activity than the mNF control in both conditions. Interestingly, Evo3 had similar hTetR activity as the mNF control, but its variability of the mCherry/BFP ratio was significantly higher, suggesting a large hTetR protein level diversity within this cell population, possibly due to the polyclonal variability of DNA amplification levels (*SI Appendix*, Fig. S3). The elevated mCherry/BFP ratios upon Dox induction in all Evo replicate populations except Evo2 further confirmed hTetR protein activity and inducibility of each replicate population, as expected.

In conclusion, we found elevated hTetR protein activity in most evolved replicate populations, except for Evo2 with the hTetR coding mutation, in agreement with the DNA amplification status and mRNA expression changes.

### Computational Modeling Uncovers Mechanisms of Protein Overexpression.

**Fig. 5.**
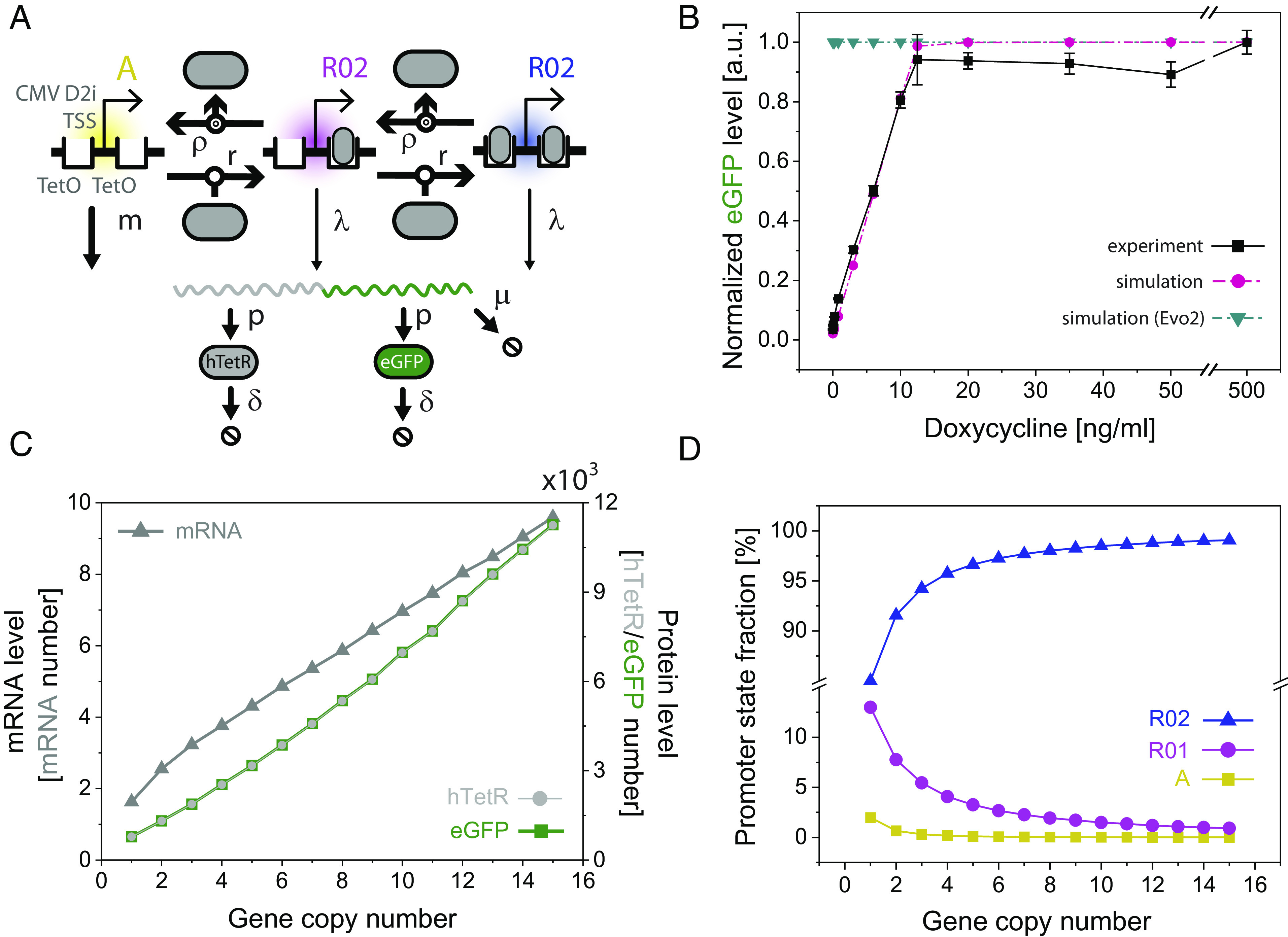
Computational modeling suggests mechanism of elevated protein expression. (*A*) Simplified chemical reaction scheme used in simulations. The unbound promoter A (thick arrow) has a high transcription rate *m*. hTetR can reversibly bind to a single (R01) or both (R02) operator sites in the promoter. Repressed promoters can still be transcribed at a low rate, leading to transcriptional leakage. (*B*) Comparison of simulated and experimental (10, 12) dose response curves (eGFP steady state levels versus Dox concentration) of ancestral CHO mNF cells, and Evo2 cells bearing inactivating hTetR mutation. (*C*) Steady state levels of eGFP and hTetR mRNA and proteins increase with increasing DNA copy number, as we observe experimentally in Evo3 through Evo6 (Fig. 4*A*). (*D*) Fraction of promoter states (unbound promoter, singly bound promoter, and doubly bound promoter) at increasing DNA copy number suggests promoter leakage as the mechanism for increased protein expression.

The increases in both DNA copy number and protein expression observed in most evolved replicates seem contradictory. How could the expression of all circuit proteins increase despite the increased expression and activity of the hTetR repressor, which should bind and block expression from their common promoter (24, 30)? To reconcile DNA amplification with increased expression of all circuit proteins, including hTetR, we adapted an earlier computational model of the mNF gene circuit in human cells (24) (Fig. 5*A* and *SI Appendix*, Fig. S4). We adjusted parameters and reactions to match the mNF dose response in ancestral CHO-mNF cells (12) (Fig. 5*B*). In this updated model, we investigated potential mechanisms for elevated steady state protein expression.

To investigate the effect of DNA amplification within the range seen experimentally in Evo3 through Evo6, we increased the amount of DNA species in the model from 1 to 15 copies in increments of 1, at a concentration of 0.05 ng/mL Dox. As seen in the experiments, we observed an increase in steady state eGFP, hTetR protein and mRNA levels (Fig. 5*C*). To investigate the cause of these increases at the level of transcriptional regulation, we looked at the promoter states at increasing DNA copy number. We observed that as DNA copy number increased, a higher percentage of promoters were in the repressed state (Fig. 5*D*). This implies that the production of mRNA is dominated by transcriptional leakage from repressed, but DNA-amplified promoters. Therefore, the model indicated that DNA amplification intensified transcriptional leakage, leading to increased mRNA, and subsequently, protein levels.

To investigate how hTetR mutation (as seen in Evo2) leads to increased expression of all circuit proteins, we reduced the hTetR-DNA binding rate to zero. We found a constant high expression of mutant hTetR and eGFP independent of inducer levels (Fig. 5*B*), consistent with the experiments. Thus, the hTetR mutation breaks the repression of gene expression in Evo2, enabling high protein expression at any Dox concentration.

Overall, computational modeling suggested that DNA amplification leads to higher expression via transcriptional leakage, which can elevate the levels of all gene circuit proteins despite the increase in hTetR protein level and repressor activity.

### TFOs Counter Drug Resistance.

The mechanisms of cancer drug resistance can be diverse, including DNA amplification (14, 15) on chromosomes or in extrachromosomal DNA (16–18), leading to overexpression of drug resistance genes (13). TFOs were recently proposed to initiate programmed cell death in oncogene amplification-driven cancers, by base-pairing with purine stretches and forming DNA triplexes within highly amplified oncogene sequences (19). However, the potential of TFOs to counter cancer drug resistance has remained unexplored. Here, we used the evolved CHO cells as a model system to test whether TFOs could also apply to treat mammalian cells that acquire drug resistance by DNA amplification. We hypothesized that targeting amplified PuroR, eGFP, or even hTetR DNA with TFOs should elicit DNA damage response and apoptosis at magnitudes that are proportional to observed fold-increases in PuroR DNA in all evolved replicate populations except for Evo1 and Evo2.

**Fig. 6.**
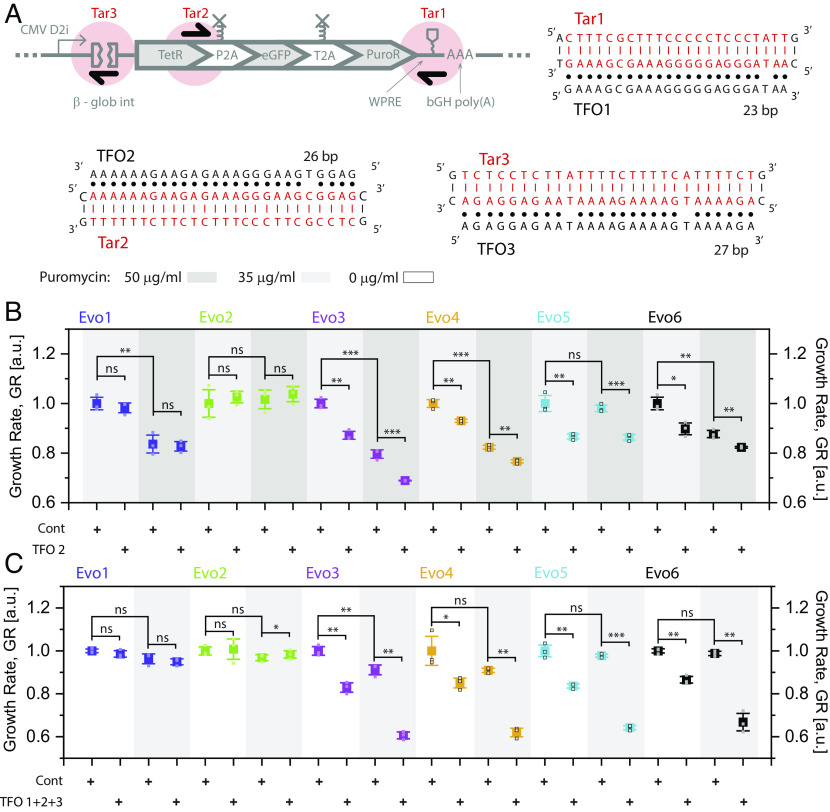
TFOs as target-specific countermeasures of drug resistance due to DNA amplification. (*A*) List of selected TFO target sites and their location within the mNF gene circuit, with the corresponding purine stretches individually highlighted. (*B*) Combinatorial treatment 35 or 50 μg/mL Puromycin and 200 nM TFO2 versus non-targeting control oligonucleotide for all evolved replicates. We normalized the growth rate for each condition by the corresponding growth rate of the control population treated with control oligo and the same concentration of Puromycin. (*C*) Combinatorial treatment of 0 or 35 μg/mL Puromycin with a total 200 nM TFOcombo mix consisting of TFO1, TFO2, and TFO3 at 1:1:1 ratio versus non-targeting control oligo, for all evolved replicates. We normalized the growth rate for each condition by the corresponding control growth rate of the control population treated with control oligo and the same concentration of Puromycin. Unpaired *t* test was performed for each comparison, *P < 0.05, **P < 0.01, ***P < 0.001, ****P < 0.0001.

To identify sequences targetable by TFOs, we searched computationally for the longest stretches of purines (A and G bases) within the mNF gene circuit. We located three potential TFO target sites (Fig. 6*A* and *SI Appendix*, Table S3) as promising candidates within the mNF gene circuit sequence. We also designed a non-targeting oligo with similar length and purine stretch to serve as control, to assess the toxicity of TFO treatment.

To test whether TFO treatment has any side effects (toxicity) in the absence of target DNA amplification, we first treated Anc5, Flp-In Parental, Evo1, and Evo2 cells with TFO3. TFO treatment did not inhibit the growth of these cell populations (*SI Appendix*, Fig. S5*A*), as expected. Next, we tested the effect of TFO3 on the remaining four replicate populations (Evo3 to Evo6). TFO3 alone did not cause significant growth inhibition, although encouragingly, some downshifts in relative growth were noticeable for all four replicates with DNA amplification (*SI Appendix*, Fig. S5*A*). Since TFO treatment efficiency increases for highly amplified DNA, we surmised that these weak TFO effects may result from evolved mNF populations that harbor a polyclonal mixture of higher and lower DNA amplification variants. Then, the variants with lower DNA amplification may be insensitive to TFO treatment and may compromise TFO efficiency. However, such low DNA amplification variants should be less drug resistant, suggesting the efficiency of TFOs may improve in combination with Puromycin treatment.

To test the efficiency of TFO+Puromycin combinatorial treatment on all DNA amplification variants, we combined TFO2 treatment with either 35 μg/mL or 50 μg/mL Puromycin. This TFO2+Puromycin combinatorial treatment suppressed significantly all four replicate populations with DNA amplification, whereas combining TFO1 or 3 with 35 μg/mL Puromycin was effective only on highly amplified replicates (Fig. 6*B* and *SI Appendix*, Fig. S5 *C* and *D*). As expected, TFO2+Puromycin (35 μg/mL or 50 μg/mL) treatment did not suppress the growth of replicates Evo1 and Evo2, which have no DNA amplification, confirming the necessity of DNA amplification for the TFO+Puromycin treatment to be effective (Fig. 6*B*). Puromycin alone inhibited minimally the evolved replicate populations, indicating sustained Puromycin resistance in each evolved population, as expected (12) (*SI Appendix*, Fig. S5*B*). In most cases, TFOs inhibited cell growth more severely in combination with 50 μg/mL Puromycin, supporting the rationale for TFO+Puromycin combinatorial treatment.

Despite the improved effectiveness of TFO+Puromycin treatment, we were still wondering whether further growth inhibition was possible, especially for samples with more modest DNA amplification like Evo4 and Evo6. Further increase of Puromycin concentration did not suppress highly amplified replicates like Evo5. We hypothesized that any single TFO would not inhibit growth sufficiently due to its target site saturation, resulting in limited DNA damage. Therefore, we reasoned that combining all TFO candidates would maximize growth inhibition by triplex formation at multiple target sites. To test this idea, we applied TFO 1, 2, and 3 together. Using this TFOcombo cocktail, we observed significant inhibition of Evo3 to Evo6 growth even without cotreatment with Puromycin (Fig. 6*C*). Combining TFOcombo with 35 μg/mL Puromycin caused up to 40% growth inhibition in all Evo3 to Evo6 replicate populations, a 25 to 30% increase relative to any single TFO+Puromycin treatment (Fig. 6*C*). Meanwhile, the lack of growth inhibition in Evo1 and Evo2 by TFOcombo confirmed that TFOs did not have side effects in the absence of target amplification.

Although Puromycin can be released from a prodrug in cancer therapies (31), its action requires cancer-specific gene overexpression, while its off-target toxicity remains poorly characterized. Therefore, we sought alternative co-treatment strategies to improve TFO efficiency. Since TFOs suppress growth by triggering DNA damage (19), we hypothesized that inhibiting the DNA repair machinery could synergize with TFO treatment. Since glycogen synthase kinase-3 (GSK3) inhibition can impair Nucleotide Excision Repair (32), we combined TFOcombo with a GSK3 inhibitor to treat all Evo replicate populations as well as control cells. By examining the physiologically safe range of GSK3 inhibitor concentration to avoid cell toxicity, we determined 500 nM as a reasonable concentration to use (*SI Appendix*, Fig. S6*A*). Pairing TFOcombo with 500 nM GSK3 inhibitor, we observed significantly stronger growth inhibition in target-amplified Evo replicate populations (*SI Appendix*, Fig. S6*B*). Thus, we identified an additional way of improving TFO efficiency.

Overall, we demonstrated that TFO treatment can serve as an effective countermeasure against drug resistance mediated by DNA amplification. TFOs could amplification-specifically sensitize highly resistant mammalian cell populations to chemotherapeutic drugs, making them strong and versatile agents in combinatorial therapies, to increase on-target effects while lowering toxic side effects.

### TFOs Overcome Drug Resistance Due to DNA Amplification in Cancer Cells.

DNA amplification is a pivotal mechanism of cancer drug resistance (33, 34). Amplification of drug resistance genes, including dihydrofolate reductase (*DHFR*) (35, 36), Multidrug Resistance Protein 1 (*MRP1* or *ABCB1*) (36), Multidrug Resistance-Associated Protein 1 (*MRP1* or *ABCC1*) (37, 38), Thymidylate Synthase (*TYMS*) (21), and Human Epidermal Growth Factor Receptor 2 (*HER2*/*ERBB2*) (2), have been identified as mediators of chemoresistance in cancer. Consequently, to test the general applicability of TFOs, we set out to investigate TFO efficacy against drug resistance in two human cancer cell line pairs that we could acquire: the Doxorubicin-resistant lung carcinoma line H69AR (20) versus its parental line H69 (ATCC) and the 5-Fluorouracil (5-FU)-resistant colon cancer line H630-R1 versus its progenitor, H630 [Edward Chu’s laboratory (21)].

**Fig. 7.**
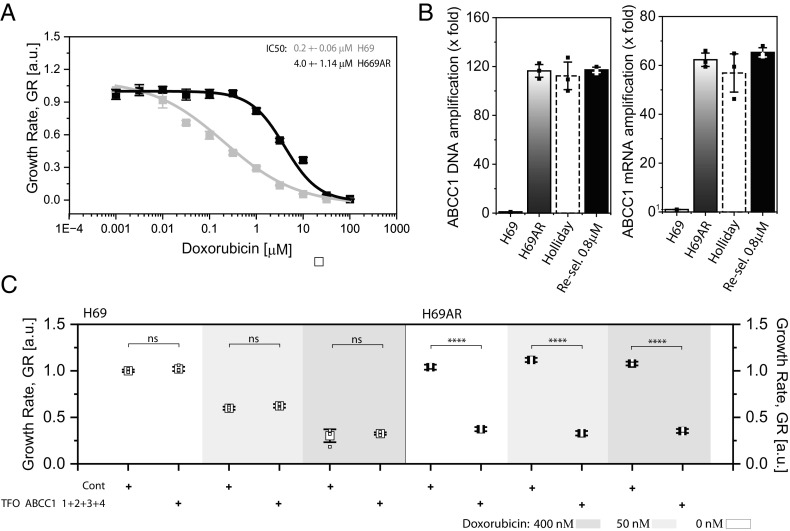
TFOs overcome DNA-amplification-dependent drug resistance in cancer cells. (*A*) Doxorubicin response curve in the Doxorubicin-resistant subline H69AR derived from H69 parental cells. We used serially diluted Doxorubicin concentrations ranging from 100 μM to 0.001 μM and normalized growth rates to the corresponding untreated controls. We determined the IC50 (Doxorubicin concentration producing 50% growth inhibition) mathematically, fitting the plot of percent growth versus logarithmic drug concentrations (n = 4 for each concentration). (*B*) *ABCC1* gene DNA and mRNA amplification in H69AR cells with or without Doxorubicin compared to H69 parental cells. We gave the cells a drug holiday by removing Doxorubicin from the growth medium for 4 wk and then re-selecting them by re-adding 0.8 μM Doxorubicin for 2 wk before qPCR (n = 3). (*C*) The effect of combinatorial treatment using 0, 50, or 400 μM Doxorubicin together with 200 nM total concentration of TFOcombo mix consisting of ABCC1_TFO1, ABCC1_TFO2, ABCC1_TFO3, and ABCC1_TFO4 at 1:1:1:1 ratio versus non-targeting control oligo, for H69AR compared with H69 parental cells. We normalized the growth rate for each condition by the growth rate of the non-treated population of each cell line. We used an unpaired *t* test for each comparison, n = 4, “ns” *P* > 0.05, *P < 0.05, **P < 0.01, ***P < 0.001, ****P < 0.0001.

To verify the drug resistance of H69AR cells, we exposed them to Doxorubicin and observed a nearly 30-fold IC50 increase compared to H69 parental cells (Fig. 7*A*). qPCR revealed an over 100-fold amplification of the *ABCC1* gene in H69AR cells relative to H69, causing a ~60-fold surge in the expression of its encoded protein MRP1 (Fig. 7*B*), as described before (34). *ABCC1* amplification remained stable in Doxorubicin-free cultures, with only a slight, non-significant dip in *ABCC1* copy number, which promptly reversed upon re-exposure to Doxorubicin (Fig. 7*B*). Therefore, Doxorubicin resistance by high *ABCC1* gene amplification is very effective and stable.

To counteract Doxorubicin resistance in H69AR cells, we designed TFOs targeting the entire ABCC1 gene region. We combined the top four TFO candidates to target ABCC1 collectively. The TFO combination had no notable impact on parental H69 cells, where ABCC1 amplification is absent (Fig. 7*C*). In contrast, the viability of H69AR cells subjected to this TFO cocktail decreased substantially relative to controls, with or without Doxorubicin (Fig. 7*C*). We observed in H630-R1 compared to H630 cells similar, but less striking effects on 5-FU resistance (*SI Appendix*, Fig. S7), as expected considering the lower level and instability of TYMS amplification along with fewer and poorer TFO target sites. Overall, these findings underscore the potential of TFOs to combat DNA-amplification-driven drug resistance in human cancer.

## Discussion

Drug resistance remains one of the greatest difficulties in cancer treatment (4), which curbs the benefits of all new approaches, including targeted therapy (2) or immunotherapy (3). It can occur in months or decades (39), and it is often associated with metastasis (40), the leading cause of mortality in cancer (41). Therefore, novel, broadly applicable strategies are needed to improve the efficiency of cancer treatment. Besides clinical relevance, understanding the adaptation of cancer cells to drugs could provide fundamental insights into cellular evolution (42). Thus, it would be important to develop experimental models of cellular adaptation that are simple enough to understand their evolutionary changes, yet sufficiently general and nonspecific to apply to many types of cancer.

We previously developed a mammalian synthetic biological model system of drug resistance evolution that should address the needs of being simple yet general (12). We engineered mammalian cells with a chromosomally integrated synthetic gene circuit that could tune the expression of the Puromycin resistance gene, *PuroR*. Thus, these cells had a small network that could mutate and evolve to address the external challenge of Puromycin treatment. By using the rodent CHO cell line, we aimed to reach results and conclusions that were sufficiently nonspecific and general, rather than pertaining to a specific human cancer type. After evolving six replicate populations of such engineered cells in constant Puromycin concentration, we repeatedly observed adaptation by elevated Puromycin expression (12). While in one population a coding mutation explained the expression change, the mechanisms of adaptation in the other five populations remained unknown.

Using qPCR, RNA-seq, and flow cytometry, we identified DNA amplification as the most common mechanism of elevated PuroR expression and thus drug resistance, despite the availability of single hTetR-disabling point mutations as easy alternatives. DNA regions surrounding PuroR were also amplified, causing mRNA and protein level changes reflecting the degree of DNA amplification. Mathematical models indicated that DNA amplification can drive up gene expression even from repressed promoters by augmenting expression leakage. Considering the frequent occurrence of DNA amplification in cancer (16, 18, 33, 43), we applied TFO therapy, which can suppress cancer cells with highly amplified DNA (19). We found that appropriately designed TFO treatment, especially when combined with the drug to which the cells have adapted, can suppress the growth of all cell populations with DNA amplification. We obtained the greatest suppression when combining the original drug with multiple TFOs targeting multiple amplified regions. This suggests a versatile strategy for addressing drug resistance due to DNA amplification, which is a frequent pathological phenotype in human cancer (43).

Drawing inspiration from TFO effects on engineered and Puromycin-adapted hamster cells, we investigated the potential of TFOs to counteract drug resistance in human cancer cell lines. We indeed found that appropriately designed TFOcombos, targeting highly amplified drug-resistance genes, can significantly impede cancer cell growth, even without chemotherapy drugs. Moreover, even cancer cells with less DNA amplification are targetable by combining chemotherapy and TFOcombo. DNA amplification in cancer manifests in various forms, including stable tandem repeats (33, 43) and less stable ecDNA (16, 18). The type of amplification may affect TFO treatment outcomes since ecDNA versus chromosomal DNA damage might differ in cytotoxicity. Hence, the underlying factors influencing TFO treatment efficiency require additional investigation.

Some questions remain open about two of the evolved replicate populations. Although both the Evo1 and Evo3 populations include some genotypes with enhancer mutations, we only found mNF gene circuit DNA amplification in Evo3 but not Evo1. The mechanism underlying elevated PuroR protein level in Evo1 cells is unclear. Since hTetR is still active in Evo1, the possibilities include post-trascriptional upregulation, or native transcriptional activation that counters hTetR repression. Another interesting aspect that requires future explanation is elevated gene expression variability in Evo3.

Synthetic biology might open new avenues to cancer treatment (44). Our methods differ from current trends to engineer various cell types to eliminate cancer (44). Instead, we combine experimental evolution (45) with synthetic biology to learn effective cancer-relevant ways to overcome drug resistance. Since cellular evolution and ecology drive the emergence, metastasis, and chemoresistance of all cancers (5), considering these factors will be crucial to achieve robust cancer treatment.

With the advent of RNA vaccines (46), nucleotide therapies are on the rise. Recently, feedback-disruptive DNA therapy was suggested for treating human viral infections (47). Our findings suggest that DNA therapy may also be relevant to treating drug-resistant cancer. Although we studied these effects in CHO cells and only two human cancer cell lines, the conservation of DNA repair mechanisms across mammals suggests that the approach could be broadly applicable to various human cancers. It will be interesting to see how the approach we developed using a synthetic biological in vitro model will perform in animal models and then possibly in the clinic for treating drug-resistant cancer.

## Materials and Methods

### Cell Culture.

The Flp-In™-CHO cell line was purchased from Invitrogen™ (Cat# R75807), then modified and evolved, while samples were frozen regularly as described previously (12). Archived cells from the “frozen record” were thawed and passaged regularly every 3 d with F-12 medium supplemented with 10% fetal bovine serum (FBS) and 1% penicillin-streptomycin. CHO ancestral mNF-PuroR replicate 5 (Anc5) and its derived Puromycin-resistant sublines were maintained in a Panasonic MCO-170AICUVL-PA cellIQ humidified incubator at 37 °C and 5% CO_2_. Evolved replicates maintained in 0.05 ng/mL Dox during the drug holiday were also supplemented with the same amount of Dox during cultivation.

The H630 parental cell line and its derived 5-FU-resistant subline H630-R1 were provided by courtesy of Ning Wei and Edward Chu (21). Both cell lines were passaged regularly every 3 to 4 d with RPMI1640 medium (Gibco™, 11875093) supplemented with 10% FBS (Corning™, 35-010-CV) and 1% penicillin-streptomycin (Gibco™, 15140122). Additionally, 1 μM 5-FU (VWR, AAA13456-14) was added to the in H630 R1 culture medium, to maintain 5-FU resistance.

The H69 parental cell line and its derived Doxorubicin-resistant subline H69AR were purchased from ATCC (HTB-119™ and CRL-11351™). The H69 parental cell line was cultured in suspension with RPMI1640 medium (Gibco™, 11875093) supplemented with 10% FBS (Corning™, 35-010-CV), 1% penicillin-streptomycin (Gibco™, 15140122), 2 mM L-glutamine (Gibco™, 25030081), 1 mM Sodium Pyruvate (Gibco™, 11360070), and 50 μM 2-mercaptoethanol (β-ME, ThermoFisher, 125472500) (37). The medium was regularly refreshed every 2 to 3 d, and cells were split every week in a 1:2 ratio. H69AR and H69 cells were maintained in the same medium, with the addition of 0.8 μM Doxorubicin (Cayman Chemical Company, 15007). The medium was refreshed every 2 d and cells were split every week at a 1:3 ratio.

### Bulk RNA Sequencing Preparation and Data Processing.

To prepare samples for RNA-seq, both CHO Evo3 and Evo4 with and without 0.05 ng/mL Dox, as well as Anc5 control cells were first seeded in 6-well plates and grown to confluency. Around 1.5 to 2 million cells from each sample were collected for total mRNA extraction. Cells were centrifuged for 5 min at 300 × g, and RNA was extracted from cell pellets using the RNeasy Plus Mini Kit (Qiagen, 74134). mRNA quality control was performed using Nanodrop, Agarose gel electrophoresis, and Agilent 2100. RNA library construction and quality control was performed by Novogene; qualified libraries were Illumina-sequenced (Novogene Corporation Inc.).

RNA sequencing reads were first quality-checked by FastQC v0.11.5. The low-quality reads (containing at least one ambiguous base, or average quality <20) were then removed using fastp v0.20.1. The clean reads were mapped to the Chinese hamster (*C. griseus* CriGri-PICRH-1.0, GCF_003668045.1) reference genome (25, 26) complemented with the synthetic gene circuit sequence using STAR 2.6.1d. The gene annotation was downloaded from the NCBI website, and read counts were obtained using FeatureCounts. Expression values were calculated and processed in R 4.1.0.z.

### DNA and mRNA Amplification Determination.

Customized qPCR primers for detecting hTetR, eGFP, PuroR, HygR, and internal reference Vinculin were designed using PrimerBlast (NCBI) and ordered from IDT and then verified for specificity and efficiency using serially diluted positive and negative CHO genomic DNA. qPCR and RT-qPCR were performed as previously described (48). Briefly, for circuit copy number detection in CHO cells, customized qPCR primers were used with PowerUp™ SYBR™ Green Master Mix (ThermoFisher Scientific, A25741). For DNA and mRNA amplification of TYMS and ABCC1 genes, corresponding TaqMan^®^ probes were purchased and performed with TaqMan^®^ Fast Advanced Master Mix (ThermoFisher Scientific, 4444557). Real copy numbers of circuit genes in every CHO clone were calculated based on the known copy number of the Vinculin reference gene in the CHO genome. TYMS and ABCC1 DNA and mRNA amplification levels were quantitated relative to the corresponding parental cells. qPCR Primer sequences and TaqMan^®^ probes are listed in *SI Appendix*, Table S2.

### CHO Transgene Analysis Using dGH In-Site™ Probe Hybridization.

Using cell preparation reagents provided by KromaTiD, BrdU and BrdC (bromodeoxynucleotide DNA nucleotide analogs) were added to live cells in culture for incorporation during DNA strand replication. Post addition, cells were allowed to grow for approximately one-half cell cycle and then metaphase formation was arrested using colcemid. Cells were then fixed in 3:1 methanol: acetic acid. From the pellet of fixed cells, metaphase spreads were then prepared on cytogenetic microscope slides using standard techniques. The prepared slides were then exposed to UV light to selectively photolyze the DNA daughter strands at the location of nucleotide analog substitutions. The photolyzed DNA strands were then degraded with an exonuclease to yield prepared dGH metaphase spreads containing only the parental, oppositely oriented DNA strands. Two distinctly colored fluorescent dGH probes were then hybridized to the target DNA sequences in the samples using standard DNA hybridization methods then the slides were further stained with DAPI to highlight the chromosomes and finally the slides washed to prepare for imaging.

The microscope slides were then scanned using a 10× objective on a Zeiss Axios automated fluorescence microscope to locate the metaphase spreads which were then reimaged using a 100× objective to produce a pool of images of metaphase spreads from each sample of the for analysis. From each pool of metaphase images, 50 metaphase spreads were selected at random, qualified for scoring. Finally, using the fluorescent signal patterns of the hybridization probes observed on the DAPI layer, the images were analyzed for the number of integrations and total chromosomes per cell.

### Humanized TetR Protein Sensor Design.

The hTetR protein sensor was designed based on pDN-D2ir-C6kwh (Addgene:44725), which contains the reporter mCherry controlled by the CMV-D2i promoter upstream. As internal copy number reference, the BFP protein-coding gene driven by an SV40 promoter was also cloned into the plasmid backbone.

### TFO Design.

To design the TFOs, we searched computationally for the longest stretches of purines (A and G bases) within the target sequences. The searches tolerated single pyrimidine gaps to lengthen and strengthen the targets, but stopped upon encountering a two-pyrimidine gap, which defined for both their 5′- and 3′-end. The 3′-amino-modified TFOs were synthesized by IDT (100 nmol, HPLC purified, lyophilized) and used after dilution in ultrapure water. All TFO sequences were designed to bind the amplified synthetic biological DNA in CHO cells and target gene sequences in cancer cells. A non-targeting control sequence was used to test for potential transfection toxicity. All TFO sequences and their target sequences are in *SI Appendix*, Table S3.

### Plasmid and Oligo Transfection.

CHO cells were seeded in 24-well plates (130,000 cells/well) and grown up to ~80% confluence (24 h) with or without Dox. 0.5 µg of the hTetR sensor vector (Fig. 4*B*) per well was transfected using Lipofectamine 3000 (Invitrogen, L3000-015; 1 µL Lipofectamine 3000: 1 µL P3000: 0.5 µg). Transfection complexes were prepared according to manufacturer protocol using OPTI MEM medium (Gibco, 31985070) as a carrier solvent. The DNA-lipid complexes were dropwise pipetted onto the cells cultured in 500 µL medium. The cells were incubated with transfection complexes for 24 h before refreshing the medium. Flow cytometry measurements were performed 48 h post transfection.

TFO was delivered onto CHO and H69AR cells with Oligofectamine (Invitrogen, 12252011) following the manufacturer’s protocol. Briefly, cells were seeded into 96-well plates (5,000 cells/well) and grown up to ~50% confluency with Dox. For each well, 1 µL of 20 µM TFO stock was diluted in 16 µL Opti-MEM (Gibco, 31985070). 0.8 µL Oligofectamine in 2.2 µL Opti-MEM was incubated for 10 min before mixing with diluted oligo. The total 20 µL mixture was then incubated at room temperature for 15 min before being dropwise pipetted onto the corresponding well with 80 µL serum-free medium. Then, 50 µL medium with 30% (v/v) FBS was added into each transfected well after 4-h incubation. Cell viability was measured 72 h post-transfection.

TFO was delivered onto H69 suspension-cultured cells with Lipofectamine 2000 (Invitrogen, 11668019) following the manufacturer’s protocol. Briefly, for each well of a 96-well plate, 160,000 cells were collected from the growth medium and resuspended in 100 µL medium without antibiotics and FBS. Then, 10 pmol of TFO combo or control oligo was diluted in 25 µL Opti-MEM. Also, 0.25 µL Lipofectamine 2000 in 25 µL Opti-MEM was incubated for 5 min at room temperature before mixing with the oligo. A total of 50 µL mixture was then incubated at room temperature for 20 min before being dropwise-pipetted onto corresponding wells with suspended H69 cells in serum-free medium. Then, 50 µL medium with 40% (v/v) FBS was added into each transfected well after 4-h incubation. Cell viability was measured 72 h post-transfection.

Cells were incubated under standard cultivation conditions (37 °C, 5% CO_2_, 90% humidity) unless stated otherwise.

### Flow Cytometry.

Flow cytometry was performed and gated consistently as previously described (48). Briefly, cells were treated with Dox or transfectants for 2 to 3 d (48 to 72 h) before flow cytometry experiments. Flow cytometry was performed using the BD LSRFortessa™ flow cytometer with High Throughput Sampler at the Stony Brook Genomics Core Facility. At least 50k gated events were collected to measure stably integrated eGFP expression and 100k gated events to measure the transiently transfected mCherry and BFP expression.

### Puromycin and GSK3 Inhibitor Treatments.

To test survival in Puromycin (Gibco, A1113803), CHO cells were seeded into 96-well plates (3,000 cells/well) and the first time point (0 h) was measured 6 h post cell seeding. Then, 35 µg/mL Puromycin was added into each well, and cell viability was measured every 24 h afterward, for 120 h post-treatment.

The GSK3 inhibitor (SB-216763, Sigma-Aldrich, S3442-5MG) 1 mM stock was prepared in DMSO, and serial-diluted in culture medium to 10,000, 1,000, 100, 10, and 1 nM concentration for cell toxicity test in 96 wells pre-seeded with 10k cells/well. For control wells, an equivalent amount of DMSO was added to test for DMSO effects. Cell viability was assayed 72 h post-treatment.

### 5-FU and Doxorubicin Cytotoxicity Assessment.

H630, H630-R1, and H69AR cells were seeded into 96-well plates (5,000 cells/well), while H69 parental cells were resuspended into 96-well plates (50,000 cells/well) prior to drug treatment. Serially diluted 5-FU or Doxorubicin was added into testing wells for a total 72 h, for H630 or H69 cells, respectively. After treatment, all testing wells were assayed for viable cells using alamarBlue™ HS Cell Viability Reagent (Invitrogen, A50100). Cells were incubated in alamarBlue reagent for 4 h. Fluorescence measurements (560-nm excitation and 590-nm emission) were taken with media blank control, using a Tecan Infinite Pro 200 (Tecan U.S. INC) plate reader/spectrophotometer. The blank intensity of culture medium at the same wavelength was subtracted from each fluorescence intensity. Relative cell viability was then calculated as the fluorescence intensity ratio:

$$R=\left(\frac{FI_{590}\;of\;the\;test\;agent}{FI_{590}\;of\;the\;control\;agent}\right).$$

IC50 (concentration causing 50% growth inhibition) was determined by data fitting to the plot of percent growth versus the logarithm of drug concentration.

### TFO Treatment and Cell Growth Inhibition Assessment.

For TFO treatment, CHO cells were seeded into 96-well plates (5,000 cells/well) and grown up to ~50% confluency prior to TFO or control oligo transfection with Oligofectamine (see details above). After 4 h of incubation, a subsequent 72-h combinatorial treatment with 35 or 50 µg/mL Puromycin or 500 nM GSK3 inhibitor was applied in testing wells. To assess growth inhibition, cell viability assays were performed using alamarBlue™ HS Cell Viability Reagent as described previously (48). Briefly, after 72 h of treatment, all samples were incubated in alamarBlue reagent for 4 h and then taken for absorbance measurement at wavelengths of 570 nm and 600 nm with media blank control using a Tecan Infinite Pro 200 (Tecan U.S. INC) plate reader/spectrophotometer. Each absorbance value was adjusted by subtracting the blank absorbance of culture medium at the same wavelength. Cell viability was measured as the alamarBlue reduction score (S) calculated as:

$$S=\left(O_{2}\times A_{570}\right)-\left(O_{1}\times A_{600}\right),$$

where $O_{1}$ and $O_{2}$ are the molar extinction coefficients of oxidized alamarBlue at 570 nm and 600 nm, respectively; and $A_{570}$ and $A_{600}$ are the absorbances of test wells at 570 nm and 600 nm, respectively. Relative growth rate was then calculated as the fold change (FC) between the average scores of the TFO-treated wells to control oligo wells.

Similarly, H630, H630-R1, and H69AR cells were seeded into 96-well plates (25,000 cells/well) and grown up to ~50% confluency prior to TFO or control oligo transfection with Oligofectamine while H69 parental cells were resuspended into 96-well plates (160,000 cells/well) prior to TFO or control oligo transfection with Lipofectamine 2000 (see details above). After 4 h of incubation, a subsequent 72-h drug treatment with 1 µM and 10 µM 5-FU or 50 nM and 400 nM Doxorubicin was applied for H630 and H69 cells, respectively. After treatment, cell viability was measured using alamarBlue™ and fluorescence readout, as in 5-FU and Doxorubicin cytotoxicity assays.

### Data Processing and Statistical Analysis.

We analyzed the flow cytometry data using FlowJo® version 10 (Becton, Dickinson and Company). We defined forward-scatter and side-scatter gates as previously described (48). We used Nikon Elements AR v4.40.00 (Build 1084) to collect and analyze imaging data, in addition to Fiji (ImageJ) and the Image Processing Toolbox in MATLAB (MathWorks, Inc.). We used MATLAB (MathWorks, Inc.), GraphPad Prism 8 (GraphPad Software), and Adobe Illustrator (Adobe, Inc.) for plotting and statistical analyses, with details described in the figure legends. We plotted results as means ± SD of three independent samples, unless the figure legend specifies otherwise.

### Computational Modeling and Mathematical Derivations.

We adapted an earlier computational model (23, 24) and adjusted the parameters and reactions to account for possible biological differences between human cells and CHO cells. To find which parameters to modify, we first adjusted all the parameters one by one and studied their effect on the dose response. We focused on a few select parameters and monitored their change using a custom MATLAB GUI. Ultimately, we changed the value of *f* (the rate of Dox entering the cell) to be 2.5x its original value. All other parameters were kept the same as in the original model. Additionally, we altered the reactions of the model to reflect the mNF system more accurately. First, we added separate translation and degradation reactions for eGFP and hTetR to reflect P2A mediated cleavage instead of a fusion. Next, we adjusted the promoter-leak reactions to occur from the bound promoters directly. With these changes, we could match the mNF dose response (10, 12). We implemented this model in MATLAB and the software Dizzy (25, 49). We then used the computational model to study the effects of parameter changes on the expression of eGFP at 0.05 ng/mL Dox. Namely, to investigate the effect of DNA amplification (Evo3 through Evo6), we changed the value of the unoccupied promoter *A* and the polymerase machinery *Aup*, ranging from 1 to 15. The initial values of all other DNA species were 0. Additionally, to investigate the effect of hTetR mutation (Evo2), we reduced to zero the value of hTetR binding to the promoter *r*. Modeling details are described in the *SI Appendix*, *Supplementary Results*.

## Supplementary Material

Appendix 01 (PDF)

## Data Availability

RNA sequencing data, SRA records, other data and computer code have been deposited in the Gene Expression Omnibus, National Center for Biotechnology Information (50) and OpenWetWare (51).
