# Supplementary material for: Adaptive DNA amplification of synthetic gene circuit opens a way to overcome cancer chemoresistance
Source: Proc Natl Acad Sci U S A. 2023 Nov 29;120(49):e2303114120. doi: 10.1073/pnas.2303114120 (PMC10710087; doi:10.1073/pnas.2303114120)
Supplement: Supplementary file 1 — Appendix 01 (PDF) [file pnas.2303114120.sapp.pdf]

PNAS

Supporting Information for:

Adaptive DNA amplification of synthetic gene circuit opens a way to overcome cancer chemoresistance

Yiming Wan[1,2,†], Quanhua Mu[3,†], Rafał Krzysztoń[1,2,†], Joseph Cohen[1,2], Damiano Coraci[1,2], Christopher Helenek[1,2], Christopher Tompkins[4], Annie Lin[1,2], Kevin Farquhar[1,2,‡], Erin Cross[4], Jiguang Wang[3], and Gábor Balázsi[1,2,*]

1 Department of Biomedical Engineering, Stony Brook University, Stony Brook, NY 11794, USA

2 The Louis and Beatrice Laufer Center for Physical and Quantitative Biology, Stony Brook University, Stony Brook, NY 11794, USA

3 Department of Chemical and Biological Engineering, Division of Life Science, State Key Laboratory of Molecular Neuroscience, The Hong Kong University of Science and Technology, Hong Kong SAR 999077, China

4 KromaTid, Inc., 1880 Industrial Cir, Ste A, Longmont, CO 80501, USA

[†] These authors contributed to the work equally.

[‡]Current affiliation: PONCE Therapeutics, Inc., 2710 Reed Road, Ste 160, Houston, TX 77051, USA.

*Corresponding author: Gábor Balázsi, Ph.D., ORCID: 0000-0002-6865-5818.

Email: gabor.balazsi@stonybrook.edu

This PDF file includes:

Supporting text

Figures S1 to S7

Tables S1 to S3

Contents

- Supporting Information Text 3
- Supplementary Results 3
  - Previous Model and Adjustments 3
  - Detailed Description of the Current Model 4
  - Mathematical Derivations 4
- Supporting Information Figures 6
- Supporting Tables 16

Supporting Information Text

Supplementary Results

Previous Model and Adjustments

We adapted the computational model of the mNF gene circuit from the 2013 Nature Communications (4:1451) paper by Nevozhay et al[1]. Briefly, the updated model described how negative regulation by hTetR impacted eGFP production at various doxycycline (Dox) concentrations. To keep the general structure of the model while accounting for potential differences between human cells and CHO cells, we sought parameter changes that would closely match the experimental dose response in the 2019 Nature Communications (10:2766) paper by Farquhar et al[2]. Specifically, we looked for the same saturation point (i.e., where the dose response stops being linear and becomes flat).

First, we altered each parameter in the model one at a time, ranging from 1/20th to 20x the original amount, to see the effect that this would have on the dose response. From this, we could find which parameters had the most drastic effects on the dose response to focus on for more fine-tuned adjustments.

From these initial findings, we further investigated these changes using a custom MATLAB GUI. Using this, we could see in real time how parameter changes impacted the dose response, and how close it was to the experimental dose response. We found that changing the value of f (the rate of dox entering the cell) to 2.5x its original value matched the dose response closely. We used this because it only involved changing one parameter for an accurate correspondence. All other parameters were kept the same as in the original model.

We also adjusted some of the reactions to make it more accurate to the current CHO system. Namely, we removed the fusion of eGFP and hTetR, and made them separate proteins, representing the use of a P2A sequence as opposed to a fusion protein. It was assumed that the translation and degradation rates of eGFP would be the same as hTetR. Additionally, the leakage reaction was changed to come from the bound promoters instead of a separate entity. All other reactions were kept the same as in the original model.

Detailed Description of the Current Model

The schematic of the current model is shown in **Fig S3.** Protein expression is driven by the promoter, where two conditions need to be met for robust transcription. First, the polymerase machinery Aup needs to be bound to the promoter. It has a binding rate α and an unbinding rate a. Second, the promoter needs to be unoccupied (*A*) by dimerized hTetR (*D*). If both of these conditions are met, there is robust transcription at a rate m (thick arrow). hTetR can bind to the promoter once (*R01*) or twice (*R02*), at a rate r, and can unbind at a rate ρ. These repressed promoters can undergo transcriptional leakage at a rate λ.

Dox can enter the cell as a zeroth order reaction at rate $f \times c$, and exits at rate f, where c is an effective inducer concentration constant multiplier. Once in the cell, Dox can bind to hTetR once (*H*) or twice (*B*) to hTetR at a rate b irreversibly. In this dox-bound state, hTetR cannot bind to the promoters, effectively leading to more time in the A state, and consequently more robust transcription.

Once mRNA is produced from transcription, it can be translated into hTetR and eGFP (*G*) protein at a rate p, or degraded at a rate μ. Consequently, these proteins can be degraded at a rate δ.

To simulate Evo3 through Evo6 (DNA Amplification), the initial values of A and Aup were changed, ranging from 1 to 15. To simulate Evo2 (TetR mutation), the rate of hTetR binding r was set to zero.

Mathematical Derivations

To complement the simulations, we also considered the differential equations to justify transcriptional leakage as the major contributor of protein expression at steady state. Let Y denote the DNA copy number.

For the polymerase machinery:

$$\frac{d[Aup]}{dt} = \alpha[Aup0] - a[Aup]$$

Additionally, by mass conservation:

$$[Aup] + [Aup0] = Y$$

Therefore, at steady state:

$$[Aup] = \frac{a}{a+\alpha}Y$$

For the promoter dynamics:

$$\frac{d[A]}{dt} = \rho[R01] - r[A][D]$$

$$\frac{d[R01]}{dt} = \rho[R02] + r[A][D] - r[R01][D] - \rho[R01]$$

$$\frac{d[R02]}{dt} = r[R01][D] - \rho[R02]$$

By conservation of mass:

$$[A] + [R01] + [R02] = Y$$

Setting these at steady state, the binding polynomial is $Q = 1 + x + x^2$, where $x = \frac{r}{\rho}[D]$.

Therefore, the probability of the promoter being in the A state is $\frac{1}{Q}$, and the probability of not being in the A state is $\frac{x+x^2}{Q}$. These are equal when $x = \frac{\sqrt{5}-1}{2}$, or when the hTetR dimer level approaches $[D]{\sim}62$. Thus, when the hTetR concentration exceeds this value, more promoters are in the bound state, and thus undergo transcriptional leakage. This effect is intensified at higher DNA copy numbers, due to the increase of hTetR protein.

The production of mRNA occurs at a rate $m[Aup][A] + \lambda([R01] + [R02])$. As $[A]$ tends towards zero, $[R01]$ + $[R02]$ tends towards Y. Thus, the production rate is approximately λY. Consequently, the steady state value becomes $\frac{\lambda}{\mu}Y$. Therefore, the steady state of mRNA is linearly proportional to the DNA copy number at high hTetR levels, which is reflected in **Fig 5D** in the main text. Similarly, the steady state of eGFP is $\frac{p\lambda}{\mu\delta}Y$, which is also linear, and reflected in **Fig. 5D** in the main text.

Supporting Information Figures

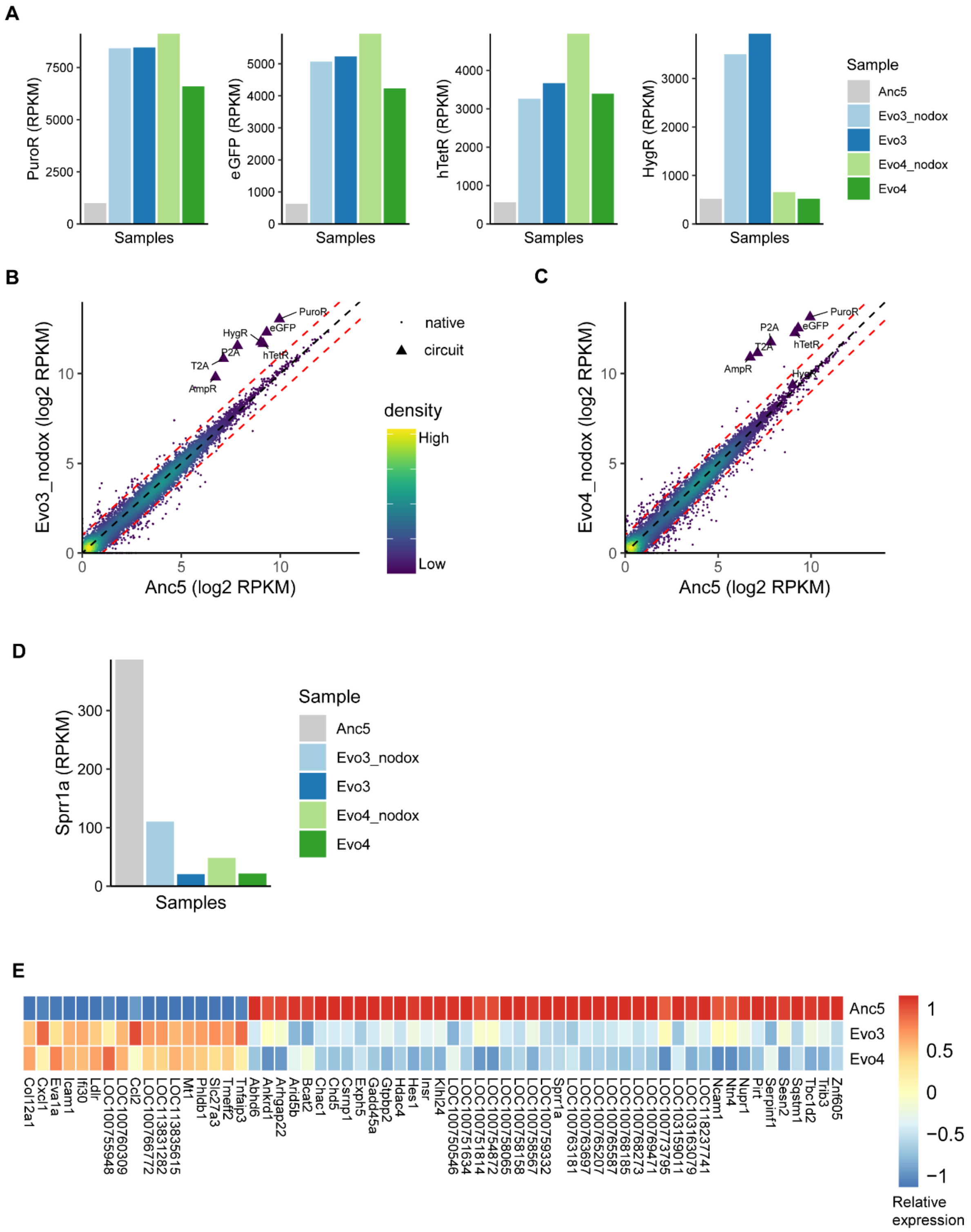

Supplementary Figure S1. Overexpression of gene circuit components in the RNA-seq data.

(A) Expression of PuroR, eGFP, hTetR, and HygR mRNA in the evolved samples compared to ancestral control (Anc5).

(B-C) Expression levels of native and circuit genes in the untreated and Puromycin-resistant mNF populations R3 (B) and R4 (C).

(D) Down-regulation of Sprr1a mRNA in the same evolved samples versus ancestral control.

(E) Heatmap showing the normalized expression of the significantly up- and down-regulated genes in the control and evolved samples.

A.

	Cell with N Inserts							Subsets of Total Inserts	
Sample	1	2	3	4	5	6	Total Inserts	HSR/Tandem	Signal on ecDNA
CHO EVO R1	17	11	1	0	1	0	47	0	1
CHO EVO R2	31	6	1	2	0	0	54	0	1
CHO EVO R3	16	16	10	6	0	0	102	50	7
CHO EVO R4	8	11	15	11	4	1	145	59	0
CHO EVO R5	19	16	8	0	1	0	80	39	0
CHO EVO R6	20	16	3	4	0	1	83	57	0
CHO NF Ctrl	31	5	1	0	0	0	44	1	0

B.

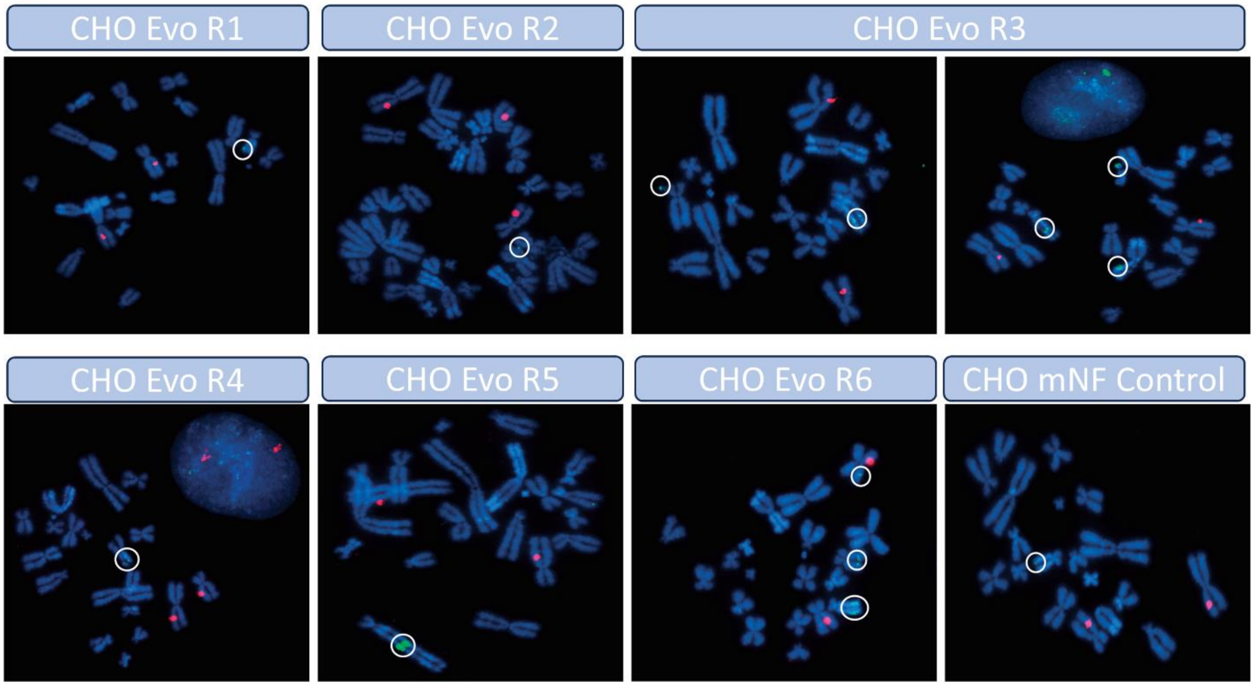

Supplementary Figure S2. DNA in-situ hybridization confirms circuit DNA amplification.

(A) Analytical summary of total detected target inserts in each CHO EVO sample as well as control Anc5 using dGH in-Site probe. Custom probes were designed to hybridize with amplified circuit DNA region, while genomic control probes were simultaneously applied for quality control. For each sample, 50 qualified images were analyzed for insert events quantification. Each counted insert was a visible fluorescence event in the chromosome, but actual DNA amplification level varies between inserts. HSR: Homogeneously Staining Regions; ecDNA: Extrachromosomal DNA.

(B) Representative DNA hybridization images for each CHO EVO and control Anc5 sample. Custom probes targeting amplified circuit DNA (Green) were mostly found in the tandem repeat/HSR format while some ecDNA events were also observed in EVO1-3 samples. Genomic control probes (Pink) were used for image quality control.

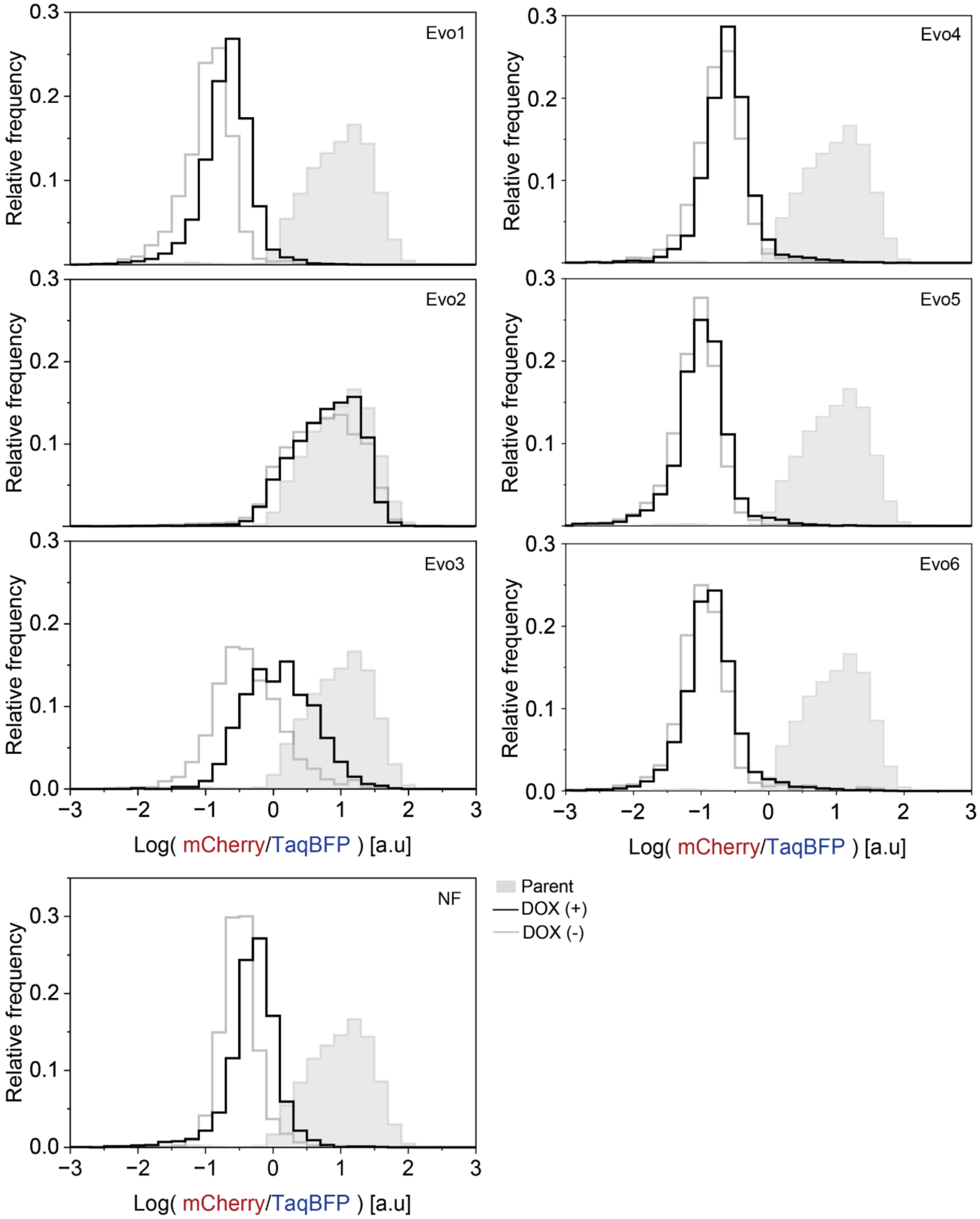

Supplementary Figure S3. Histograms of hTetR activity measured by the mCherry/BFP ratio at the single-cell level. Averaged data corresponding to the histograms is in **Fig. 4C**.

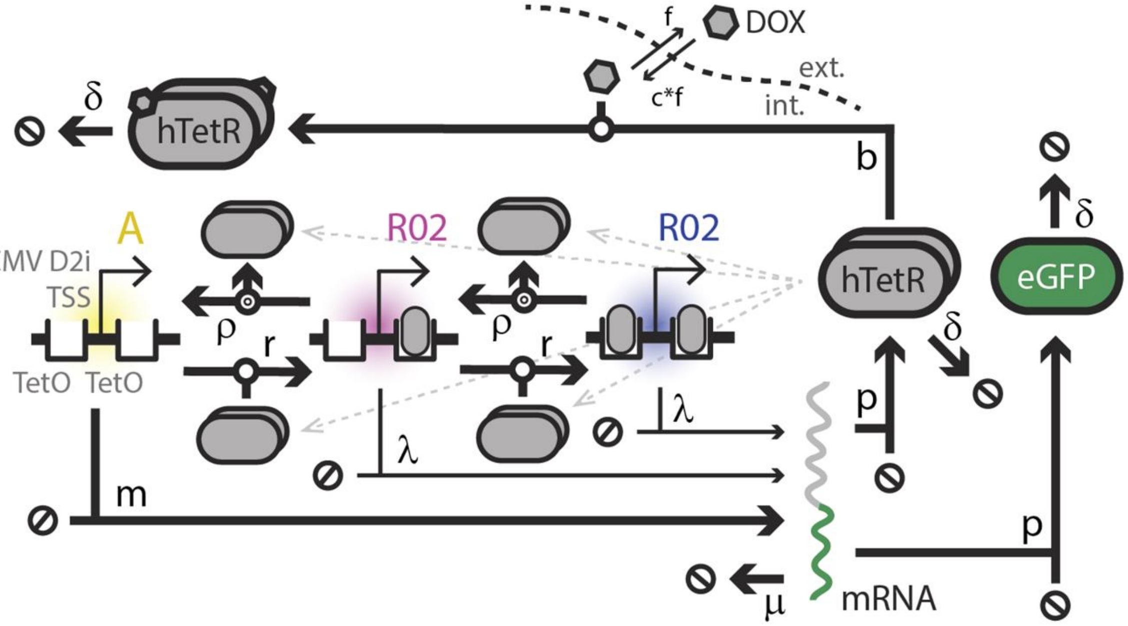

Supplementary Figure S4. Detailed chemical reaction scheme of the mNF gene circuit model used in the simulations.

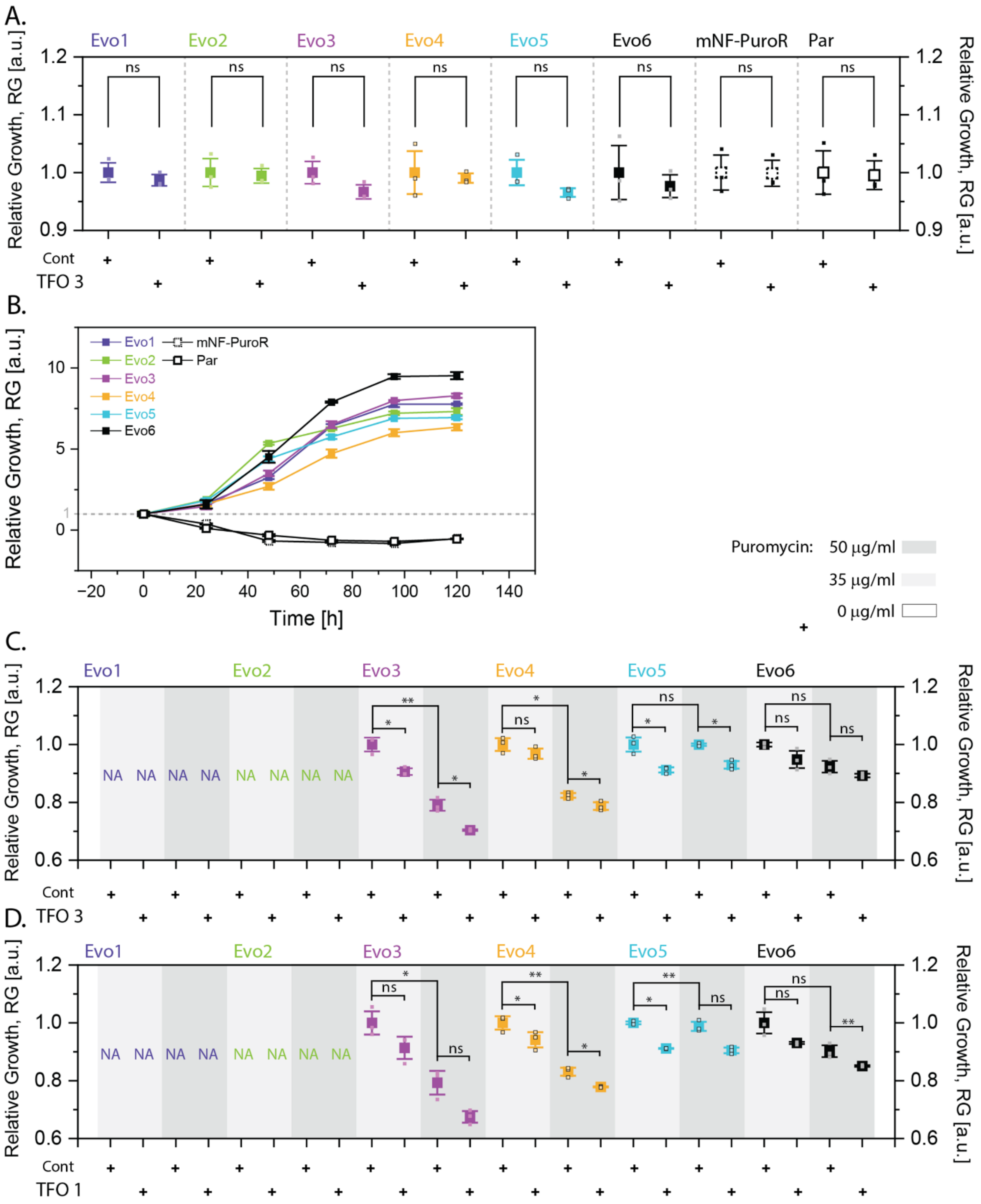
A.
Evo1
Evo2
Evo3
Evo4
Evo5
Evo6
mNF-PuroR
Par
ns
Relative Growth, RG [a.u.]
1.2
1.1
1.0
0.9
Cont
TFO 3
B.
Evo1
Evo2
Evo3
Evo4
Evo5
Evo6
mNF-PuroR
Par
Relative Growth, RG [a.u.]
10
5
1
0
−20
0
20
40
60
80
100
120
140
Time [h]
Puromycin:
50 μg/ml
35 μg/ml
0 μg/ml
C.
Evo1
Evo2
Evo3
Evo4
Evo5
Evo6
NA
Relative Growth, RG [a.u.]
1.2
1.0
0.8
0.6
Cont
TFO 3
D.
Evo1
Evo2
Evo3
Evo4
Evo5
Evo6
NA
Relative Growth, RG [a.u.]
1.2
1.0
0.8
0.6
Cont
TFO 1

Supplementary Figure S5. Combining TFO with Puromycin treatment enhances growth inhibition.

(A) Effect of TFO3 treatment on all evolved mNF replicates as well as the mNF control sample Anc5 and a parental sample.

(B) Sustained Puromycin resistance in all evolved mNF replicates compared to the mNF control sample Anc5 and parental sample.

(C) Effect of 35 or 50 µg/ml Puromycin combined with 200 nM TFO3 or non-targeting control oligo for all the evolved replicates. The growth rate for each condition was normalized by the corresponding control population treated with control oligo and 35 µg/ml Puromycin. Unpaired t-test was performed for each comparison, *P<0.05, **P<0.01, ***P<0.001.

(D) Effect of 35 or 50 µg/ml Puromycin combined with 200 nM TFO1 or non-targeting control oligo for all the evolved replicates. Th growth rate for each condition was normalized to the corresponding control population treated with control oligo and 35 µg/ml Puromycin. Unpaired t-test was performed for each comparison, *P<0.05, **P<0.01, ***P<0.001.

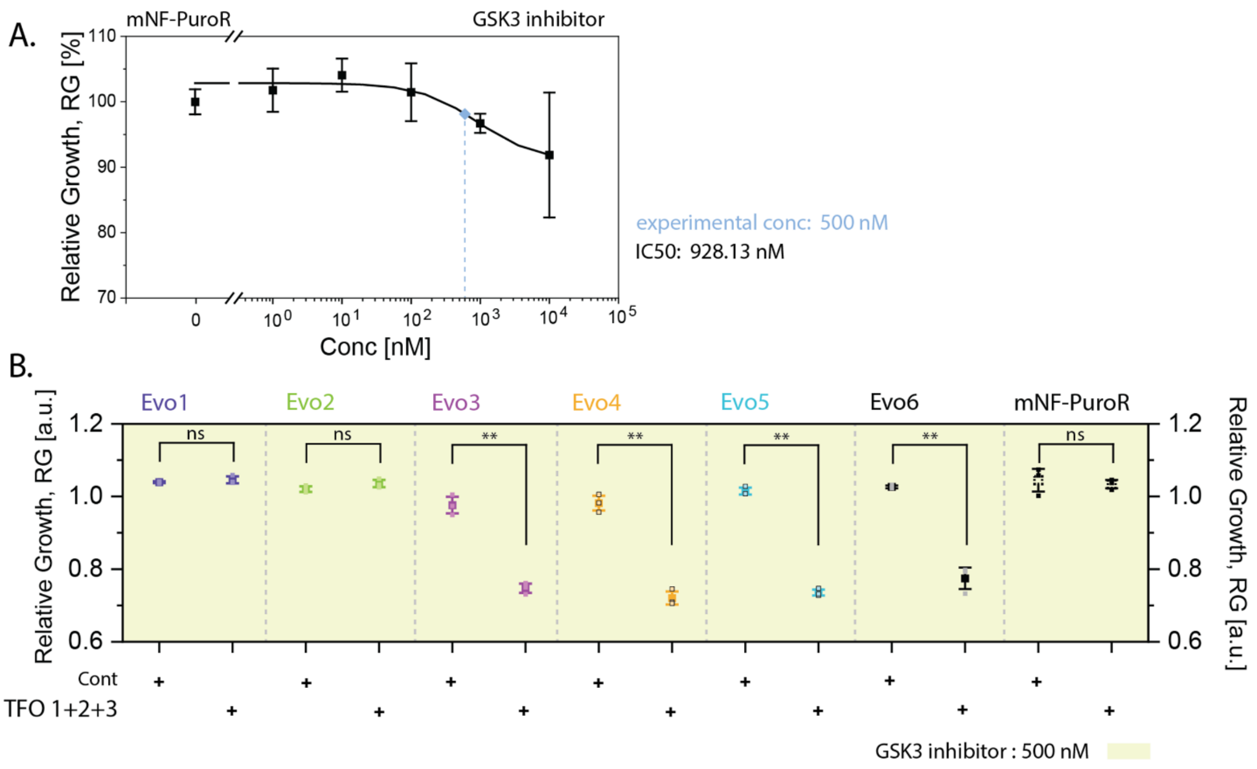

Supplementary Figure S6. Cotreatment with GSK3 inhibitor improves target-specific growth inhibition by TFO treatment.

(A) Determining the physiological safe range of GSK3 inhibitor by its titration on mNF control sample Anc5. We selected 500 nM concentration of GSK3 inhibitor as the maximum safe dose for combinatorial treatment with TFO.

(B) Effect of cotreatment by a total of 200 nM TFOcombo (TFO1, 2 and 3 at 1:1:1 ratio) or non-targeting control oligo and 500 nM GSK3 inhibitor on all the evolved replicates. The growth rate of each condition was normalized to the corresponding control population treated with control oligo and 500 nM GSK3 inhibitor. Unpaired t-test was performed for each comparison, *P<0.05, **P<0.01, ***P<0.001.

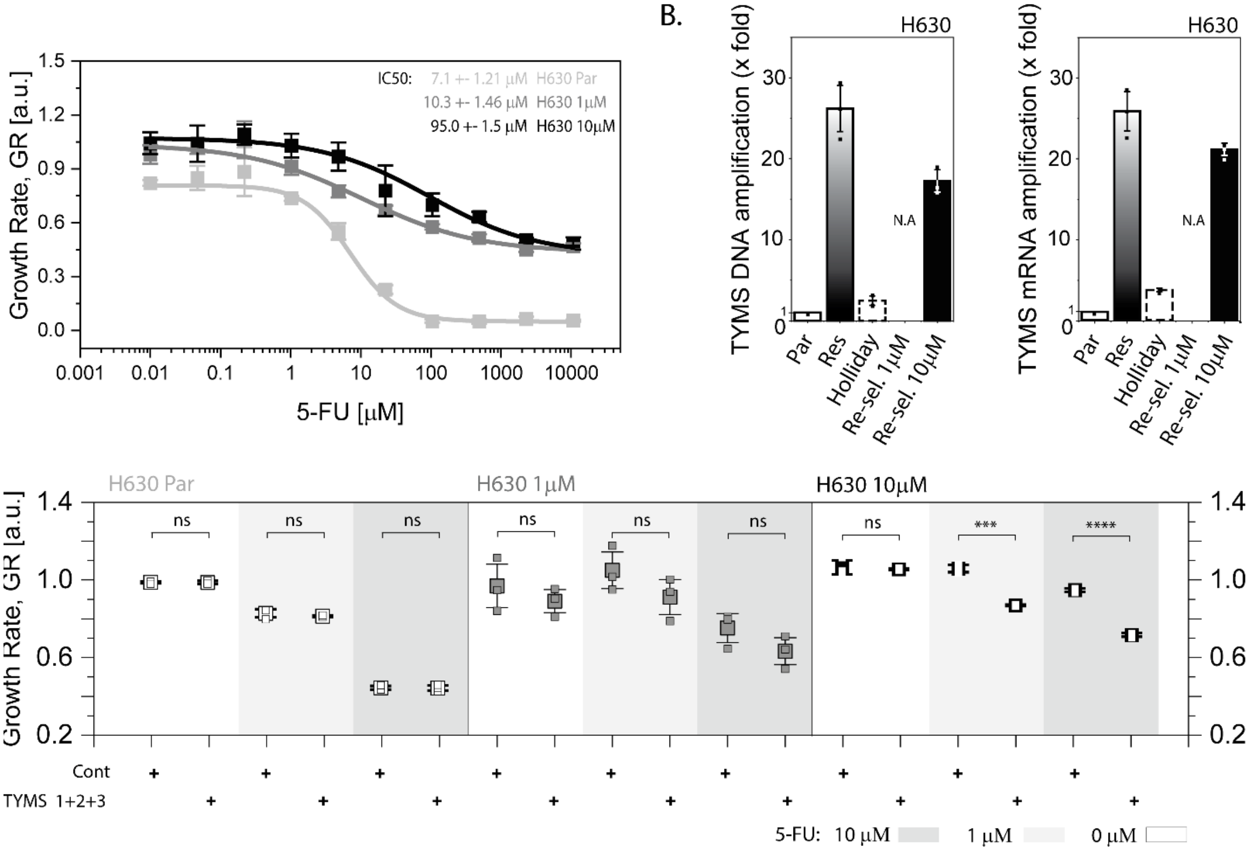

Supplementary Figure S7. TFOs synergize with chemotherapy to overcome drug-resistance in cancer cells with moderate DNA amplification.

(A) 5-Fluorouracil (5-FU) response curve in the 5-FU-resistant subline H630-R1 derived from H630 parental cells maintained in 10 μM and 1 μM 5-FU, respectively. We used serially diluted 5-FU concentrations ranging from 10000 μM to 0.01 μM and normalized growth rates to the corresponding untreated controls. We determined the IC50 (5-FU concentration producing 50% growth inhibition) mathematically, fitting the plot of percent growth versus logarithmic drug concentrations (n = 3 for each concentration).

(B) TYMS gene DNA and mRNA amplification in H630-R1 cells with or without 5-FU presence, compared to H630 parental cells. We gave the cells a drug holiday was by removing 5-FU from the growth media for 4-weeks and then re-selecting them by re-adding 10 μM 5-FU for 2-weeks before qPCR (n = 3).

(C) The effect of combinatorial treatment using 0, 1, or 10 μM 5-FU together with 200 nM total TFOcombo mix consisting of TYMS_TFO1, TYMS_TFO2 and TYMS_TFO3 at 1:1:1 ratio versus non-targeting control oligo, for H630-R1 compared to H630 parental cells. We normalized the growth rate for each condition by the growth rate of the untreated population. We used an unpaired t-test for each comparison, n = 4 for H630 parental cells and 10 μM 5-FU reselected H630-R1 cells, and n = 3 for 1 μM 5-FU reselected H630-R1 cells. 'ns' P>0.05, ***P<0.001, ****P<0.0001.

Supporting Tables

Supplementary Table S1 Fit parameters from **Fig. 4A**.

	**Dox -**	**Dox +**
**intercept**	148.81 ± 75.11	228.02± 64.48
**slope**	29.48 ± 10.73	26.27 ± 7.99
**Pearson's r**	0.81	0.85

Supplementary Table S2 Lists of primer sequences and TaqMan probes used for copy number detection and gene expression analysis.

Primer	Source	Sequence / Identifier
PuroR qPCR primer forward	Farquhar, Charlebois et al. 2019	CGAGTACAAGCCCACGGT
PuroR qPCR primer reverse	Farquhar, Charlebois et al. 2019	AGTTCTTGCAGCTCGGTGA
eGFP qPCR primer forward	This study	CGAAGGCTACGTCCAGGAGC
eGFP qPCR primer reverse	This study	CCTCGAACTTCACCTCGGCG
HygR qPCR primer forward	This study	ACGTTGCAAGACCTGCC
HygR qPCR primer reverse	This study	GATTCCTTGCGGTCCGAATG
hTetR qPCR primer forward	This study	AATGGGTTCTAGACTGGACAAGAGCAA
hTetR qPCR primer reverse	This study	AAGTCTTGCCATGACTCGCC
Vinculin qPCR primer forward	Farquhar, Charlebois et al. 2019	GCTGGTTGCTAAGAGGGAGG
Vinculin qPCR primer reverse	Farquhar, Charlebois et al. 2019	ATCAGAGGCAGCTTTCACGG
TYMS TaqMan Copy Number Assay	Life Technologies Corporation	CAT#4331182, Assay ID: Hs00560134_cn
TYMS TaqMan Gene Expression Assay	Life Technologies Corporation	CAT#4331182, Assay ID: Hs00426586_m1
ABCC1 TaqMan Copy Number Assay	Life Technologies Corporation	CAT#4331182, Assay ID: Hs01754544_cn
ABCC1 TaqMan Gene Expression Assay	Life Technologies Corporation	CAT#4331182, Assay ID: Hs01561483_m1

Supplementary Table S3 Lists of Triplet-forming Oligo sequences used for cell treatment. Mismatched thymine (T) is labeled in red and non-interacting thymine (T) is labeled in blue.

TFO Oligo	Target sequence (5'-3')	Oligo Sequence (3'-5')
TFO1	AATAGGGAGGGGGAAAGCGAAAG	AATAGGGAGGGGGAAAGTGAAAG
TFO2	AAAAAAGAAGAGAAAGGGAAGCGGAG	AAAAAAGAAGAGAAAGGGAAGTGGAG
TFO3	AGAAAATGAAAAGAAAATAAGAGGAGA	AGAAAATGAAAAGAAAATAAGAGGAGA
ABCC1_TFO1	AAGGAGGAGGAGGAAGGGGAGGGG (Intron 1)	AAGGAGGAGGAGGAAGGGGAGGGG
ABCC1_TFO2	AATAGGCAAGAAGGAAGGGAGAAGGAAGAAGG (Intron 1)	AATAGGTAAGAAGGAAGGGAGAAGGAAGAAGG
ABCC1_TFO3	GGGAGGAAGAGGGAAAGGAATGA (Intron 5)	GGGAGGAAGAGGGAAAGGAATGA
ABCC1_TFO4	AAGAAAAGGGAAAGAAGAGAAATAAGA (Intron 1)	AAGAAAAGGGAAAGAAGAGAAATAAGA
TYMS_TFO1	AATAAAGAAAAAGAGAAGAA (Intron 3)	AATAAAGAAAAAGAGAAGAA
TYMS_TFO2	GTGGGGTGGGAGGAGGGGGGAGGGATAG (Intron 3)	GTGGGGTGGGAGGAGGGGGGAGGGATAG
TYMS_TFO3	GGAAAGGAAAGAGGAAAGGACGA (Intron 1)	GGAAAGGAAAGAGGAAAGGATGA
Control	None	GAAGTTTCTCCAGCCGGCCCAACAAC

References

1 Nevozhay, D., Zal, T. & Balazsi, G. Transferring a synthetic gene circuit from yeast to mammalian cells. *Nat Commun* **4**, 1451, doi:10.1038/ncomms2471 (2013).

2 Farquhar, K. S. *et al.* Role of network-mediated stochasticity in mammalian drug resistance. *Nat Commun* **10**, 2766, doi:10.1038/s41467-019-10330-w (2019).
